# Polydatin and Nicotinamide Prevent Iron Accumulation and Lipid Peroxidation in Cellular Models of Mitochondrial Diseases

**DOI:** 10.3390/antiox14020215

**Published:** 2025-02-13

**Authors:** Paula Cilleros-Holgado, David Gómez-Fernández, Rocío Piñero-Pérez, José Manuel Romero-Domínguez, Diana Reche-López, Mónica Álvarez-Córdoba, Ana Romero-González, Alejandra López-Cabrera, Marta Castro De Oliveira, Andrés Rodríguez-Sacristán, Susana González-Granero, José Manuel García-Verdugo, José Antonio Sánchez-Alcázar

**Affiliations:** 1 Centro Andaluz de Biología del Desarrollo (CABD-CSIC-Universidad Pablo de Olavide), 41013 Sevilla, Spain; pcilhol@upo.es (P.C.-H.); dgomfer1@acu.upo.es (D.G.-F.); rpieper@alu.upo.es (R.P.-P.); jmromdom@upo.es (J.M.R.-D.); dreclop@alu.upo.es (D.R.-L.); malvcor@upo.es (M.Á.-C.); aromgon1@upo.es (A.R.-G.); alopcab2@alu.upo.es (A.L.-C.); 2 Neuropediatria, Neurolinkia, C. Jardín de la Isla, 8, Local 4 y 5, 41014 Sevilla, Spain; martadecastro@neurolinkia.com; 3 FEA Pediatría, Centro Universitario Hospitalar de Faro, R. Leão Penedo, 8000-386 Faro, Portugal; 4 Neuropediatría, Servicio de Pediatría, Hospital Universitario Virgen Macarena, 41009 Sevilla, Spain; arodriguezsacristan@us.es; 5 Departamento de Farmacología, Radiología y Pediatría de la Facultad de Medicina de la Universidad de Sevilla, 41009 Sevilla, Spain; 6 Laboratory of Comparative Neurobiology, Cavanilles Institute of Biodiversity and Evolutionary Biology, University of Valencia and CIBERNED-ISCIII, 46980 Valencia, Spain; susana.gonzalez@uv.es (S.G.-G.); j.manuel.garcia@uv.es (J.M.G.-V.)

**Keywords:** mitochondrial diseases, *GFM1*, iron accumulation, lipid peroxidation, ferroptosis, mtUPR, direct reprogramming

## Abstract

Ferroptosis, an iron-dependent form of non-apoptotic cell death, is regulated by a complex network involving lipid metabolism, iron homeostasis, and the oxidative-reductive system, with iron accumulation and lipid peroxidation as key drivers. Mitochondrial dysfunction and ROS overproduction often underlie the pathogenesis of mitochondrial diseases, for which treatment options are limited, emphasizing the need for novel therapies. In this study, we investigated whether polydatin and nicotinamide could reverse ferroptosis-related pathological features in cellular models derived from patients with pathogenic *GFM1* variants. Mutant fibroblasts showed increased iron and lipofuscin accumulation, altered expression of iron metabolism-related proteins, elevated lipid peroxidation, and heightened susceptibility to erastin-induced ferroptosis. Treatment with polydatin and nicotinamide effectively corrected these alterations and reduced iron accumulation and lipid peroxidation in induced neurons. Furthermore, chloramphenicol treatment in control cells mimicked the mutant phenotype, suggesting that these pathological changes are linked to the mitochondrial protein synthesis defect characteristic of pathogenic *GFM1* variants. Notably, adding vitamin E to the polydatin and nicotinamide co-treatment resulted in a reduction in the minimum effective concentration, suggesting potential benefits of its inclusion. In conclusion, the combination of polydatin, nicotinamide, and vitamin E could represent a promising therapeutic option for patients with mitochondrial disorders caused by pathogenic *GFM1* variants.

## 1. Introduction

Ferroptosis has been characterized as an iron-dependent, non-apoptotic form of cell death predominantly driven by lipid peroxidation [1]. Recent studies, however, have revealed that ferroptosis is regulated by an intricate network involving lipid metabolism, iron homeostasis, and redox balance, with iron accumulation and lipid peroxidation identified as primary inducers [2,3].

Iron is essential for cellular processes such as oxygen transport, myelin biosynthesis [4], neurotransmitter synthesis [5], collagen formation [6], mitochondrial electron transport chain (mtETC) component formation [7], and energy metabolism. Furthermore, iron is central to innate immunity [8]. While low iron levels can impair erythropoiesis and lead to anemia, excessive iron is toxic due to the generation of reactive oxygen species (ROS) through the Fenton reaction [9]. Therefore, iron metabolism is tightly regulated, balancing storage and transport to maintain homeostasis. The dysregulation of iron metabolism, leading to its accumulation, may result from increased uptake or diseases affecting its regulation [10].

Iron uptake varies depending on the cell type. Generally, ferric iron (Fe^3+^) binds to transferrin in the blood, forming a transferrin–iron complex that is internalized via endocytosis upon binding to transferrin receptor protein 1 (TfR1). Within endosomes, Fe^3+^ is released and reduced to ferrous iron (Fe^2+^) by the metalloreductase six-transmembrane epithelial antigen of prostate 3 (STEAP3). Fe^2+^ is then transported into the cytoplasm via divalent metal transporter 1 (DMT1), contributing to the labile iron pool (LIP). Cytoplasmic iron can be stored in ferritin (Ft) in its ferric form, exported from the cell through ferroportin (FPN), or transported into mitochondria by mitoferrin1 (Mfrn1) or mitoferrin2 (Mfrn2). In mitochondria, iron serves various purposes, such as heme or Fe-S cluster biosynthesis, or it can be stored in mitochondrial ferritin (Mt-Ft) [11].

The degradation of cytoplasmic ferritin, a process termed ferritinophagy, is regulated by the nuclear receptor coactivator 4 (NCOA4) and contributes to ferroptosis [12]. Indeed, iron accumulation in the cytoplasm promotes ROS formation via the Fenton reaction, triggering lipid peroxidation, which is a crucial step in ferroptosis [13].

Lipid peroxidation occurs through both enzymatic and non-enzymatic mechanisms. Enzymatic peroxidation involves lipoxygenases acting on arachidonic and linoleic acids to produce hydroxylated phospholipids. Other enzymes, such as cytochrome P450 and cyclooxygenases, also contribute to lipid endoperoxide formation [13]. In contrast, non-enzymatic lipid peroxidation is driven by radicals through three stages: initiation, propagation, and termination. During initiation, free radicals react with polyunsaturated fatty acids, forming lipid radicals. In the propagation step, oxygen reacts with these lipid radicals, creating peroxyl radicals that can further react with other lipids, thereby perpetuating a chain reaction. Termination occurs when radicals react to form stable, non-radical species [14].

High levels of cholesterol and polyunsaturated fatty acids esterified in phospholipids in cellular membranes render cells particularly susceptible to lipid peroxidation. This compromises membrane integrity and function, disrupts cellular signaling and metabolism, heightens oxidative stress, and ultimately leads to ferroptosis [15,16].

However, iron accumulation and lipid peroxidation are not the only drivers of ferroptosis. System Xc-inhibition, reduced glutathione peroxidase 4 (GPX4) activity, and the depletion of reduced glutathione (GSH) also contribute to this regulated cell death pathway [2].

Additionally, ferroptosis differs from other forms of regulated cell death in its effects on mitochondrial morphology. Features such as smaller, denser mitochondria, reduced or absent cristae, and fragmented outer mitochondrial membrane (OMM) are observed during ferroptosis [17]. Moreover, since mitochondria are the primary sources of ROS, they are closely involved in ferroptosis by promoting lipid peroxidation [18].

Under healthy conditions, ferroptosis plays an important role in maintaining cellular homeostasis. However, dysfunctional ferroptosis has been associated with several diseases, including cancer, neurodegenerative diseases, inflammatory disorders, and mitochondrial DNA depletion syndrome [19].

Mitochondrial diseases, which are characterized by mitochondrial dysfunction and ROS overproduction [20], result from mutations in either nuclear DNA (nDNA) or mitochondrial DNA (mtDNA) and affect approximately 1 in 5000 individuals, classifying them as rare diseases [21]. Symptoms vary widely, ranging from common issues, such as neurodegeneration and exercise intolerance, to less frequent manifestations, such as diabetes mellitus [22]. While many mitochondrial diseases arise from mutations in genes encoding mtETC proteins, mutations in genes involved in mitochondrial maintenance, transcription, or translation also occur. For instance, mutations in the nuclear gene *G elongation factor mitochondrial 1* (*GFM1*), which encodes the mitochondrial translation elongation factor G1 (EF-G1), cause combined oxidative phosphorylation deficiency type 1, an autosomal recessive disorder which manifests as dystonia, feeding difficulties, and severe encephalopathy, among other symptoms [23]. Currently, most mitochondrial diseases have only symptomatic or palliative treatments, highlighting the urgent need for novel therapeutic approaches.

Our laboratory previously demonstrated that the combination of polydatin and nicotinamide holds promise as a therapeutic option for pathogenic *GFM1* variant-related mitochondrial diseases by activating the mitochondrial unfolded protein response (mtUPR) [24]. This combination was essential, as neither compound alone supported the survival of mutant cells in galactose medium, highlighting their synergistic effect [24]. Moreover, similar effects have been observed with other compounds, such as tetracyclines [25] and pterostilbene in combination with mitochondrial enhancers in several pathogenic variants causing mitochondrial diseases [26].

In the present study, we show that polydatin and nicotinamide prevent intracellular iron accumulation, lipid peroxidation, and ferroptosis in fibroblasts and induced neurons derived from patients with pathogenic *GFM1* variants. Our findings suggest that mitochondrial dysfunction due to impaired mitochondrial protein synthesis drives iron and lipofuscin accumulation, lipid peroxidation, and increased ferroptosis sensitivity. Furthermore, adding vitamin E to polydatin and nicotinamide therapy may reduce the minimum effective concentration, thereby potentially enhancing the precision and efficacy of clinical treatments for patients with these debilitating mitochondrial disorders.

## 2. Materials and Methods

### 2.1. Reagents

Prussian Blue (03899), paraformaldehyde (PFA) (158127), nicotinamide (N7004), D-glucose (G7879), dimethyl sulfoxide (DMSO) (17093), and Luperox^®^ (168521) were purchased from Sigma-Aldrich (Saint Louis, MO, USA).

Phosphate-buffered saline (PBS) (102309) was obtained from iNtRON Biotechnology (Seongnam, Republic of Korea). Anti-TfR1 (13-6800), anti-iron sulfur cluster assembly scaffold protein (ISCU) (MA5-26595), anti-Mfrn2 (PA5-42498), anti-mitochondrial acyl carrier protein (mtACP) (PA5-89967), anti-GPX4 (MA5-32827), anti-SIRT3 (PA5-13222), propidium iodide (PI) (11539226), Mitotracker^TM^ Red CMXRos (M46752), Mitotracker^TM^ Deep Red FM (M22426), Mitotracker^TM^ Green FM (M7514) 4′,6-diamino-2-phenylindole (DAPI) (D1306), bovine serum albumin (BSA) (BP9702), Hoechst (10150888), MitoSOX^TM^ (M36008), and Bodipy^®^ 581/591 C11 (D3861) were obtained from Invitrogen^TM^/Molecular probes (Eugene, OR, USA)/ThermoFisher Scientific (Waltham, MA, USA).

Anti-iron response protein 1 (IRP1) (sc-166022), anti-ferritin light chain (sc-74513), anti-FPN (sc-518125), anti-Tau (sc-32240), D-galactose (sc-202564), chloramphenicol (sc-3594), and deferiprone (sc-211220) were purchased from Santa Cruz Biotechnology (Dallas, TX, USA).

Anti-DMT1 (ab55735), anti-Mt-Ft (ab124889), anti-NFS1 cysteine desulfurase (NFS1) (ab58623), anti-LYR motif-containing protein 4 (LYRM4) (ab253001), anti-frataxin (FXN) (ab219414), anti-NCOA4 (ab86707), anti-pantothenate kinase 2 (PANK2) (ab119070), anti-EF-G1 (ab173529), anti-MtCO2 (ab79393), anti-MnSOD (ab68155), anti-Nrf2 (ab62352), Goat Anti-Rabbit IgG H&L (HRP) (ab6721), Rabbit Anti-Mouse IgG H&L (HRP) (ab6728), and Rabbit Anti-Goat IgG H&L (HRP) (ab6741) were purchased from Abcam (Cambridge, UK).

Anti-actin antibody (MBS448085) was obtained from MyBioSource (San Diego, CA, USA). Polydatin (21246) was purchased from Cayman-Chemicals (Ann Arbor, MI, USA). MitoPeDPP^®^ (M466) and Mito-FerroGreen (M489) were purchased from Dojindo Molecular Technologies, Inc. (Rockville, MD, USA).

### 2.2. Ethical Statements

The ethics committees of the Hospital Universitario Virgen Macarena and Hospital Universitario Virgen del Rocío (Seville, Spain) approved the current study (Mitocure Code 0543-N-16, dated 11 October 2016), in accordance with the principles of the Declaration of Helsinki and the International Conferences on Harmonisation and Good Clinical Practice Guidelines.

### 2.3. Fibroblast Culture

We cultured fibroblasts derived from skin biopsies of five mitochondrial patients (P1, P2, P3, P4, and P5) who carried the following pathogenic variants:

- P1 and P2 (siblings): heterozygous variant in exon 2 at c.179C>G, p.(Thr60Ser), and in exon 17 at c.2068C>T, p.(Arg690Cys) of the *GFM1* gene.
- P3: heterozygous variant at c.1404delA, p.(Gly469Valfs*84) in exon 12, and c.2011C>T, p.(Arg671Cys) in exon 16 of the *GFM1* gene.
- P4: heterozygous variant in exon 10 at c.1297_1300del, p.(Asp433Lysfs*20), and in exon 16 at c.2011C>T, p.(Arg671Cys) of the *GFM1* gene.
- P5: heterozygous variant in exon 15 at c.1822C>T, p.(Arg608Trp), and in exon 16 at c.2011C>T, p.(Arg671Cys) of the *GFM1* gene.

Primary human skin fibroblasts from age- and sex-matched healthy volunteers were also cultured as control cells (C1, C2, and C3). The ages of the patients were as follows: P1 (13 years), P2 (11 years), P3 (9 years), P4 (13 years), and P5 (12 years). The control subjects were C1 (13 years), C2 (12 years), and C3 (9 years). All experiments were performed using cells with a passage number below 10.

Control and patient cells were obtained in accordance with the 1964 Declaration of Helsinki (amended in 2001). Fibroblasts were cultured in Dulbecco’s Modified Eagle’s Medium (DMEM) (Gibco^TM^, Waltham, MA, USA) at 37 °C with 5% CO_2_, supplemented with 10% Fetal Bovine Serum (FBS) (Gibco^TM^, Waltham, MA, USA) and 1% penicillin/streptomycin (Sigma-Aldrich, Saint Louis, MO, USA).

### 2.4. Treatments

Cells were treated with 10 µM polydatin and 10 µM nicotinamide. These concentrations were based on previous experiments conducted in our laboratory, in which cells were cultured in a nutrient-restrictive medium containing galactose as the unique carbon source. In those experiments, cells were treated with various concentrations of polydatin and nicotinamide (1, 5, 10, 25, 50, and 100 µM). From these trials, 10 µM was selected as the optimal concentration, as it was the minimum concentration required to ensure the survival of mutant cells in galactose medium [24]. Importantly, no toxic effects were observed at higher concentrations, as cells remained viable under these conditions. This aligns with previous studies, such as those demonstrating the beneficial effects of nicotinamide on mitigating accelerated aging in fibroblasts derived from patients with progeroid syndromes, even at concentrations as high as 8 mM [27]. Moreover, polydatin at concentrations up to 100 µM has been reported to reduce inflammasome activation in fibroblasts exposed to paraquat, further confirming its positive effects without toxicity at concentrations higher than those used in this study [28]. Regarding human toxicity thresholds, nicotinamide has a reported toxic dose of 6 g (approximately 44 mM), which is far greater than the concentration used in this study [29]. Similarly, the toxic dose of polydatin has been established at 80 mg, corresponding to approximately 206 µM, which is significantly higher than the 10 µM concentration utilized in our experiments [30].

In addition to the primary treatments, the control cells were treated with 10 µM chloramphenicol to mimic the mitochondrial protein synthesis deficiency observed in patient-derived fibroblasts. For specific assays, additional controls were included: P2 cells treated with 50 µM vitamin E served as a negative control for superoxide anion and cellular lipid peroxidation measurements, while C1 cells treated with 500 µM Luperox^®^ acted as a positive control for cellular lipid peroxidation detection. Furthermore, P2 cells treated with 100 µM deferiprone were used as a negative control in the following assays: Prussian Blue staining, lipofuscin accumulation (detected by autofluorescence), mitochondrial iron detection, cellular and mitochondrial lipid peroxidation, GSH levels measurement, and sensitivity to erastin-induced ferroptosis assays. This helped demonstrate the dependence of these processes on iron accumulation. Similarly, C1 cells treated with 100 µM deferiprone served as a negative control in LIP detection.

Finally, to evaluate potential synergistic effects, cells were treated with a combination of 1 µM polydatin, nicotinamide, and vitamin E.

### 2.5. Determination of Iron Accumulation

Iron accumulation was assessed via Perl’s Prussian Blue staining in fibroblasts and induced neurons (iNs), as previously described by our group [31,32]. Images were acquired using an Axio Vert A1 fluorescence microscope (Zeiss, Oberkochen, Germany) and analyzed with Fiji-ImageJ software version 1.53.2.

Iron content was further quantified by inductively coupled plasma mass spectrometry (ICP-MS) using an Agilent 7800 mass spectrometer (Agilent Technologies, Santa Clara, CA, USA). Cell extracts for ICP-MS were prepared via acid digestion with nitric acid.

Mitochondrial iron content was measured using Mito-FerroGreen. Cells were seeded in µ-Slide 4 Ibidi plates in DMEM glucose medium for three days. After that, cells were washed three times with Hank’s Balanced Salt Solution (HBSS) (21-022-CV, Corning, NY, USA) and incubated with 100 nM Mitotracker^TM^ Deep Red FM for 45 min at 37 °C to visualize the mitochondrial network. Cells were then washed four times with HBSS and incubated with 5 µM Mito-FerroGreen for 30 min at 37 °C. Following this, cells were washed three times with HBSS. Images were acquired using a DeltaVision system with an Olympus IX-71 fluorescence microscope (Applied Precision; Issaqua, WA, USA) and analyzed with Fiji-ImageJ software version 1.53.2.

### 2.6. Determination of Lipofuscin Accumulation

Lipofuscin accumulation was determined via autofluorescence using a Nikon A1R confocal microscope (Nikon, Tokyo, Japan). Emission spectra were obtained using confocal laser scanning microscopy and measured in 20 lipofuscin granules across 30 cells. Autofluorescence intensity was quantified using Fiji-ImageJ software version 1.53.2.

### 2.7. TEM Analysis

Cells were seeded in 8-well Permanox chamber slides (177380, ThermoFisher Scientific, Waltham, MA, USA) in DMEM glucose medium for three days. After that, cells were fixed with 3.5% glutaraldehyde in 0.1 M phosphate buffer for 10 min at 37 °C. After fixation, cells were post-fixed in 2% OsO4, rinsed, dried, and embedded in Durcupan Resin (44610-1EA, Sigma-Aldrich (Saint Louis, MO, USA)). Using a diamond knife, ultrathin pieces of 70 nm were cut and examined using a transmission electron microscope (FEI Tecnai G2 Spirit BioTwin) equipped with a Xarosa digital camera (20 Megapixel resolution) and Radius image acquisition software version 2.1 (EMSIS GmbH, Münster, Germany).

### 2.8. Determination of Labile Iron Pool (LIP)

To determine the LIP levels, cells were seeded in 12-well plates. After three days, cells were incubated in HBSS with 20 mM HEPES and 0.25 µM Calcein-AM at 37 °C for 15 min. Following this, cells were washed twice with HBSS and then incubated in HBSS supplemented with 20 mM HEPES, 150 Mm NaCl, and 5 mM glucose for 10 min. Cells were subsequently incubated with 500 µM deferiprone for 10 min. Following the addition of deferiprone, a first fluorescence measurement was taken using a POLARstar Omega Microplate Reader (BMG Labtech, Offenburg, Germany). After 1 h of incubation with the chelator, a second fluorescent measurement was taken using the same microplate reader. The ratio of the second fluorescence measurement to the first was used as the LIP value. Moreover, the results were normalized to the protein content of each sample.

### 2.9. Immunoblotting

Western blot analysis was conducted using standard protocols. Following protein transfer, nitrocellulose membranes (1620115, Biorad, Hercules, CA, USA) were blocked in 5% BSA in TTBS (blocking solution) and incubated with primary antibodies at a dilution range of 1:500 to 1:2000 at 4 °C overnight. After that, membranes were incubated for one hour at room temperature with the matching secondary antibody conjugated to horseradish peroxidase (HRP) at a dilution range of 1:2500–1:10,000. The Immun Star HRP substrate kit (1705061, Biorad, Hercules, CA, USA) was used to visualize protein bands using the Chemidoc^TM^ MP Imaging System (Biorad, Hercules, CA, USA). To standardize the acquired data, we used the housekeeping actin protein and the mean expression levels of control cells. For proteins that were sufficiently separated, membranes were divided, and each section was incubated with a distinct antibody. Finally, the processed membranes were analyzed using ImageLab^TM^ software version 6.1 (Biorad, Hercules, CA, USA).

### 2.10. Determination of Lipid Peroxidation

Lipid peroxidation was determined using the fluorescent probe 4,4-difluoro-5-(4-phenyl-1,3-butadienyl)-4-bora-3a,4a-diaza-s-indacene-3-undecanoic acid (Bodipy^®^ 581/591 C11). Cells were seeded in 6-well plates and, after three days, incubated with 5 µM Bodipy^®^ 581/591 C11 for 30 min at 37 °C. Control cells were treated with 500 µM Luperox^®^ for 20 min, which served as a positive control. Images were acquired using an Axio Vert A1 fluorescence microscope (Zeiss, Oberkochen, Germany) and analyzed using Fiji-ImageJ software version 1.53.2.

Mitochondrial lipid peroxidation was determined using the fluorescent probe [3-(4-phenoxyphenylpyrenylphosphino)propyl]triphenylphosphonium iodide (MitoPeDPP) (MitoPeDPP^®^). Fibroblasts were incubated with 300 nM MitoPeDPP^®^ and 100 nM Mitotracker^TM^ Deep Red FM for 45 min. After that, cells were stained with DAPI at 1 µg/µL for 5 min. Images were taken using a DeltaVision system with an Olympus IX-71 fluorescence microscope (Applied Precision; Issaqua, WA, USA) and analyzed using Fiji-ImageJ software version 1.53.2.

### 2.11. Determination of Mitochondrial Superoxide Anion

To determine mitochondrial superoxide anion generation, cells were seeded in DMEM glucose medium for 72 h. After that, cells were washed twice with PBS 1× and stained with 100 nM Mitotracker^TM^ Green FM and 5 µM MitoSOX^TM^ Red and 100 nM MitoTracker^TM^ DeepRed FM for 45 min at 37 °C. Subsequently, cells were washed with PBS 1× and incubated with 1 ug/mL DAPI for 5 min. Finally, images were acquired using a DeltaVision system with an Olympus IX-71 fluorescence microscope (Applied Precision; Issaqua, WA, USA) and analyzed using Fiji-ImageJ software version 1.53.2.

### 2.12. Cell Transfection with Human GFM1 Plasmid

To perform cDNA complementation assays, we introduced FLAG-tagged human *GFM1* cDNA (HG20851-CF, SinoBiological, Beijing, China) into C2 and P2 cells. Initially, cells were seeded in 6-well plates in DMEM glucose medium with 10% FBS, without antibiotic supplementation, and cultured for three days. Subsequently, cells were transfected with Lipofectamine^®^ 2000 (11668027, ThermoFisher Scientific, Waltham, MA, USA) and FLAG-tagged human *GFM1* cDNA in Opti-MEMTM I Reduced Serum Medium (31985062, ThermoFisher Scientific, Waltham, MA, USA) for 24 h. The anti-DYKDDDDK tag antibody (A00187) used for detection was obtained from GenScript (Piscataway, NJ, USA).

### 2.13. Immunofluorescence Microscopy

For immunofluorescence microscopy, cells were seeded in DMEM glucose medium for 72 h. Then, cells were fixed with 4% PFA for 10 min and permeabilized with 0.01% Triton X-100 for an additional 10 min. Subsequently, cells were incubated with 5% donkey serum (ab7475, Abcam, Cambridge, UK) for 1 h. After that, cells were incubated with the primary antibody at an appropriate dilution range (1:100–1:400) in 5% donkey serum overnight at 4 °C. The following day, cells were washed twice with PBS 1× and incubated with the corresponding secondary antibody diluted 1:200 in 5% donkey serum for 2 h at room temperature. Cells were then washed twice with PBS 1× and stained with DAPI 1 µg/mL for 10 min. Finally, the cells were washed with PBS 1× five times. Images were acquired using a DeltaVision system with an Olympus IX-71 fluorescence microscope (Applied Precision; Issaqua, WA, USA) and analyzed by Fiji-ImageJ software version 1.53.2.

### 2.14. Determination of Reduced Glutathione

To determine the reduced glutathione (GSH) levels, cells were seeded in DMEM glucose medium for 72 h. After that, cells were washed twice with PBS 1× and stained with 20 µM ThiolTracker^TM^ Violet and 30 min at 37 °C. Subsequently, cells were washed with PBS 1× and fixed with 4% PFA for 10 min. Finally, images were acquired using a DeltaVision system with an Olympus IX-71 fluorescence microscope (Applied Precision; Issaqua, WA, USA) and analyzed using Fiji-ImageJ software version 1.53.2.

### 2.15. Direct Reprogramming

Using direct reprogramming, we generated induced neurons (iNs) from both mutant and control fibroblasts [33,34,35]. Cells were initially seeded in µ-Slide 4 Ibidi plates in DMEM + Glutamax medium (10566016, ThermoFisher Scientific, Waltham, MA, USA) supplemented with 10% FBS and 1% penicillin/streptomycin for 24 h. Cells were then infected at a multiplicity of infection (MOI) of 30 with a single lentiviral vector which contained two shRNAs against the REST complex and two neural linage-specific transcription factors, as previously described [36]. The plasmids were a gift from Dr. Malin Parmar (Developmental and Regenerative Neurobiology, Lund University, Lund, Sweden). The following day, the medium was replaced with fresh DMEM + Glutamax, and 48 h later, the neural differentiation medium (supplemented NDiff27 Y40002, Takara-Clontech, San Jose, CA, USA) was added. Every two to three days, half of the neural differentiation medium was replenished. After 18 days of cellular infection, the medium was replaced with NDiff27 supplemented only with growth factors. On day 21, cells were treated with polydatin and nicotinamide at 10 µM for seven days. On day 28 post-infection, neuronal purity and conversion efficiency were determined, identifying Tau^+^ cells as iNs, and all experiments were assessed accordingly.

### 2.16. Determination of Induced Ferroptosis Sensitivity

To determine sensitivity to induced ferroptosis, erastin, which is a well-characterized ferroptosis inducer, was used. First, the cells were seeded in 96-well plates at a density of 8 × 10^3^ cells/well in DMEM glucose medium 24 h. The following day, nuclei were stained with 1 µg/mL Hoechst for 10 min at 37 °C. Subsequently, the wells were washed twice with PBS 1× and incubated with propidium iodide (PI) at 1 µM in HBSS, along with erastin at 5 µM in those wells where ferroptosis induction was desired. An automated follow-up of the number of nuclei stained with PI was performed using a CellDiscoverer7 fluorescence microscope (Zeiss Carl AG, Oberkochen, Germany). Images of the wells were acquired with a 5× objective over 25 h at 1-h intervals at 37 °C and 5% CO_2_. Image analysis was performed using Fiji-ImageJ software version 1.53.2.

### 2.17. Statistical Analysis

For datasets with sample sizes greater than 30, parametric statistical methods, particularly one-way ANOVA, were used to evaluate statistical differences among more than two groups. For sample sizes below 30, non-parametric methods were applied, such as the Mann–Whitney test for comparing two groups and the Kruskal–Wallis test for comparing multiple groups. All statistical analyses were performed using GraphPad Prism 9.4.1 (GraphPad Software, San Diego, CA, USA), with a significance level set at *p* ≤ 0.05.

## 3. Results

### 3.1. Polydatin and Nicotinamide Reduce ROS Production in Mutant GFM1 Fibroblasts

**Figure 1:**
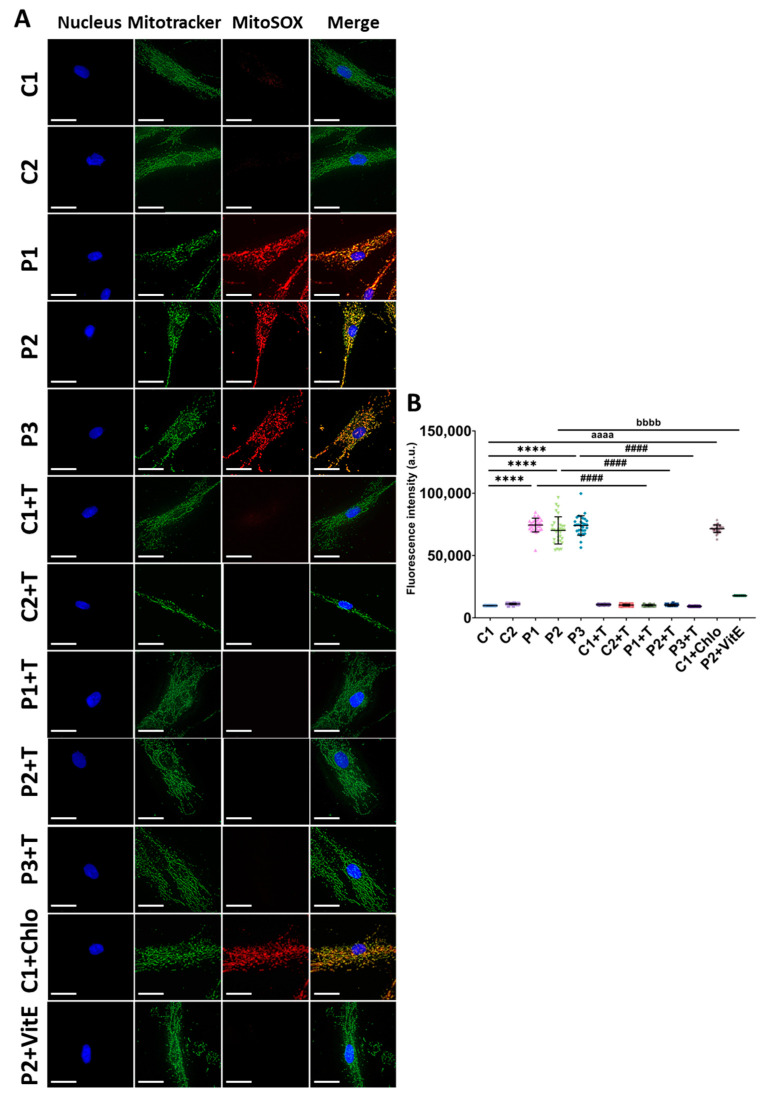
Superoxide anion production in untreated and treated (+T) control (C1 and C2) and patient (P1, P2, and P3) cells. Cells were treated with polydatin and nicotinamide for seven days. P2 cells treated with 50 µM vitamin E (P2+VitE) served as a negative control. C1 cells were treated with 10 µM chloramphenicol (C1+Chlo) to mimic the mitochondrial protein synthesis deficiency observed in patients’ fibroblasts. (**A**) Representative images of MitoSOX^TM^ staining. Cells were also stained with Mitotracker^TM^ Green FM to visualize the colocalization of both probes. Scale bar: 20 µm. (**B**) Quantification of fluorescence intensity. Data represent the mean ± SD of three independent experiments (at least 30 images were taken from each condition and experiment). Number of biological replicates = 3. **** *p* < 0.0001 between control and mutant fibroblasts. ^####^ *p* < 0.0001 between untreated and treated patients’ fibroblasts. ^aaaa^ *p* < 0.0001 between untreated and chloramphenicol-treated C1 cells. ^bbbb^ *p* < 0.0001 between untreated and vitamin E-treated P2 cells. a.u.: arbitrary units. Refer to Supplementary Figure S1 for comparison between grouped data of controls and patients.

First, given the established link between mitochondrial dysfunction and the overproduction of ROS in mitochondrial diseases, we measured superoxide anion levels using the MitoSOX^TM^ probe. As expected, fibroblasts derived from patients displayed significantly higher fluorescence intensity compared to control fibroblasts, indicating elevated superoxide anion levels. Treatment with polydatin and nicotinamide successfully reduced this fluorescence intensity. Similarly, control fibroblasts treated with 10 µM chloramphenicol, which mimics the mitochondrial protein synthesis defect observed in patients’ fibroblasts, exhibited higher fluorescence intensity compared to untreated control fibroblasts. Furthermore, P2 cells were treated with 50 µM vitamin E, a well-characterized antioxidant, as a negative control (Figure 1).

### 3.2. Polydatin and Nicotinamide Correct Iron and Lipofuscin-like Aggregates Accumulation in Mutant GFM1 Fibroblasts

**Figure 2:**
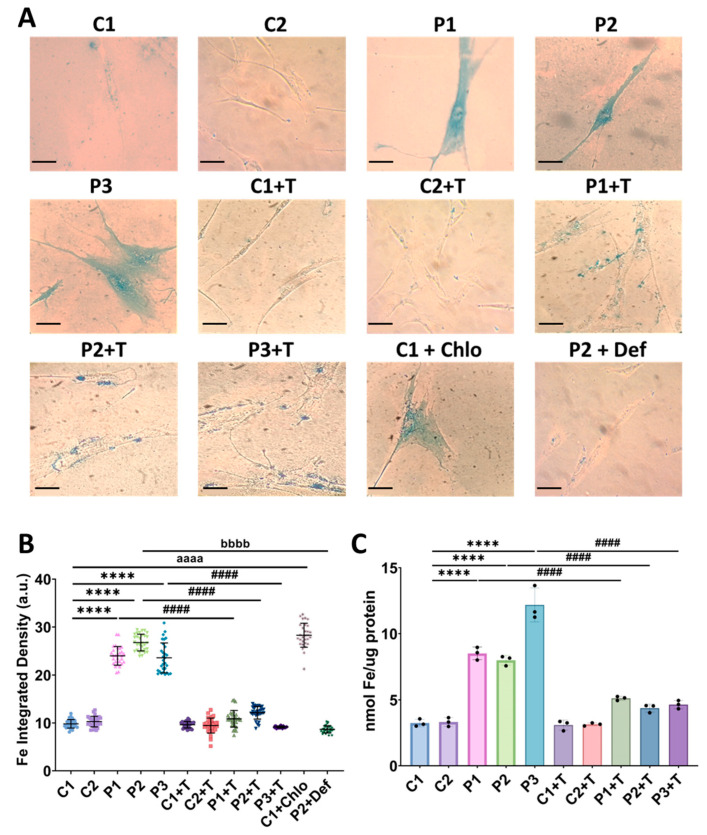
Intracellular iron accumulation in untreated and treated (+T) control (C1 and C2) and patient (P1, P2, and P3) cells. Cells were treated with polydatin and nicotinamide for seven days. P2 cells were treated with 100 µM deferiprone (P2+Def) to confirm the specificity of Prussian Blue staining for iron. C1 cells were treated with 10 µM chloramphenicol (C1+Chlo) to mimic the mitochondrial protein synthesis deficiency observed in patients’ fibroblasts. (**A**) Representative images of Prussian Blue staining. Scale bar: 20 µm. (**B**) Quantification of iron integrated density. (**C**) Iron content measured by ICP-MS. Data represent the mean ± SD of three independent experiments (at least 30 images were taken from each condition and experiment). Number of biological replicates = 3. **** *p* < 0.0001 between control and mutant fibroblasts. ^####^ *p* < 0.0001 between untreated and treated patients’ fibroblasts. ^aaaa^ *p* < 0.0001 between untreated and chloramphenicol-treated C1 cells. ^bbbb^ *p* < 0.0001 between untreated and deferiprone-treated P2 cells. a.u.: arbitrary units. Refer to Supplementary Figure S2 for comparison between grouped data of controls and patients.

Then, as superoxide anions can damage iron–sulfur cluster proteins and release Fe^2+^, which can react with ROS via the Fenton reaction to produce more toxic species [37], we next analyzed intracellular iron accumulation in both control and mutant *GFM1* fibroblasts using Prussian Blue staining. Our results revealed a significant increase in iron staining in patients’ fibroblasts compared to control cells. To confirm the specificity of Prussian Blue staining for iron, we treated P2 fibroblasts with 100 µM deferiprone, an iron chelator. Notably, polydatin and nicotinamide supplementation significantly reduced iron accumulation in mutant *GFM1* fibroblasts. Additionally, C1 cells treated with 10 µM chloramphenicol, a mitochondrial protein synthesis inhibitor, exhibited intracellular iron accumulation, suggesting that the mitochondrial translation deficiency characteristic of patients’ fibroblasts is responsible for this iron accumulation (Figure 2A,B). Furthermore, to assess whether rescuing the mutant gene reduces the observed intracellular iron levels, we introduced a FLAG-tagged human *GFM1* cDNA into both control and patients’ cells. As expected, mutant fibroblasts expressing the wild-type cDNA showed a significant reduction in intracellular iron accumulation, with levels comparable to those in control cells (Figure S3). To further corroborate the abnormal intracellular iron content, we measured iron levels using ICP-MS. Mutant *GFM1* cells exhibited a significant increase in total iron content, which was effectively reduced by polydatin and nicotinamide treatment (Figure 2C).

**Figure 3:**
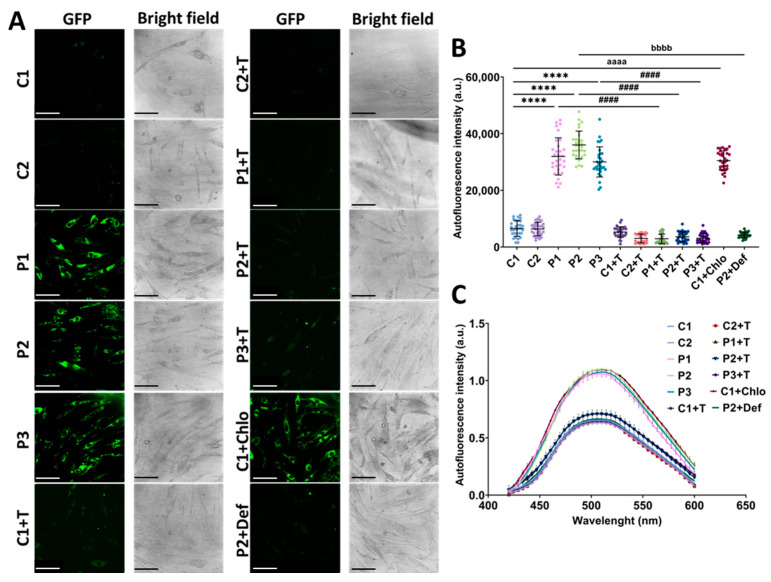
Autofluorescence analysis of untreated and treated (+T) control (C1 and C2) and patient (P1, P2, and P3) cells. Cells were treated with polydatin and nicotinamide for seven days. C1 cells treated with 10 µM chloramphenicol (C1+Chlo) were used to simulate the mitochondrial protein synthesis deficiency observed in patients’ cells. P2 cells treated with 100 µM deferiprone (P2+Def) served as a negative control to confirm the iron-dependence of lipofuscin-like granules. (**A**) Representative images showing GFP and bright field channels of control and mutant *GFM1* fibroblasts. Scale bar: 100 µm. (**B**) Autofluorescence spectra of lipofuscin granules with excitation laser at 405 nm. (**C**) Quantification of autofluorescence intensity. Data represent the mean ± SD of three independent experiments (at least 30 images were taken from each condition and experiment). Number of biological replicates = 3. **** *p* < 0.0001 between control and mutant fibroblasts. ^####^ *p* < 0.0001 between untreated and treated patients’ cells. ^aaaa^ *p* < 0.0001 between untreated and chloramphenicol-treated C1 fibroblasts. ^bbbb^ *p* < 0.0001 between untreated and deferiprone-treated P2 cells. a.u.: arbitrary units. Refer to Supplementary Figure S4 for comparison between grouped data of controls and patients.

Next, as reported in other conditions with intracellular iron accumulation [31,38,39], this trace element is typically accumulated in the form of lipofuscin. To determine whether iron was accumulating within lipofuscin in mutant *GFM1* fibroblasts, we assessed lipofuscin accumulation using autofluorescence and electron microscopy. Interestingly, patients’ cells showed an increase in autofluorescence intensity compared to control fibroblasts. Polydatin and nicotinamide treatment markedly reduced the autofluorescence intensity in mutant cells. Additionally, treating P2 cells with 100 µM deferiprone reduced autofluorescence intensity, indicating that the accumulation of lipofuscin-like material is iron-dependent. To confirm the lipofuscin-like characteristics of the autofluorescent material, we performed confocal laser scanning microscopy of lipofuscin granules, observing an emission peak at 520–540 nm, consistent with the characteristics of lipofuscin granules reported in retinal pigment epithelium cells of the eye [40]. Moreover, treatment of control cells with 10 µM chloramphenicol to mimic the mitochondrial protein synthesis failure described in the patients’ cells, resulted in an increase in the autofluorescence intensity, comparable to that observed in patients’ fibroblasts (Figure 3).

**Figure 4:**
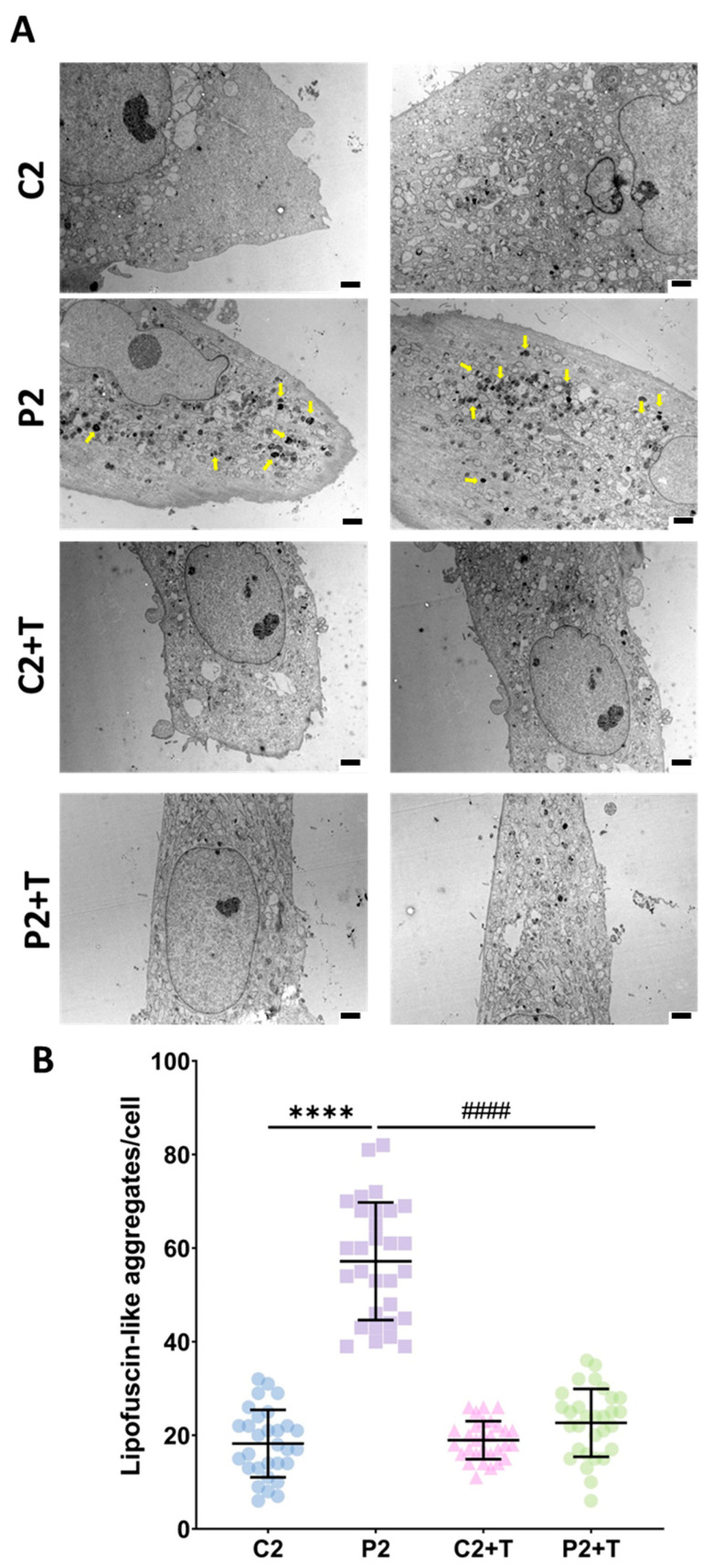
Electron microscopy images of untreated and treated (+T) control (C2) and patient (P2) cells. Treatment with polydatin and nicotinamide was applied for seven days. (**A**) Representative electron microscopy images. Scale bar: 2 µm (**B**) Quantification of lipofuscin-like aggregates per cell Data represent the mean ± SD of three separate experiments (at least 30 images were taken for each condition and experiment). Number of biological replicates = 3. **** *p* < 0.0001 between control and patient cells. ^####^ *p* < 0.0001 between untreated and treated P2 fibroblasts. Yellow arrows: lipofuscin-like granules.

Electron microscopy analysis further confirmed the accumulation of lipofuscin-like aggregates in P2 fibroblasts, as the number of intracellular lipofuscin-like granules was significantly higher in patient’s cells compared to control fibroblasts. Remarkably, supplementation with polydatin and nicotinamide reduced the number of lipofuscin-like granules in P2 fibroblasts (Figure 4). In addition, higher magnification of these images provided detailed views of the lipofuscin-like granules, revealing their formation inside mitochondria and subsequent release from these organelles into the cytosol (Figure S5).

### 3.3. Polydatin and Nicotinamide Correct Protein Expression Levels of Proteins Involved in Iron Metabolism and Reduce Mitochondrial Iron Content in Mutant GFM1 Fibroblasts

Next, we performed Western blot analysis of proteins involved in iron metabolism. This analysis included proteins regulating iron metabolism, such as IRP1; iron uptake proteins, including TfR1, DMT1, and Mfrn2; iron storage proteins, such as Ft and Mt-Ft; iron exportation protein FPN; iron–sulfur cluster biosynthesis proteins, FXN, LYRM4, ISCU, and NFS1; and ferritinophagy protein NCOA4. Additionally, we measured the expression levels of proteins implicated in coenzyme A metabolism, given the importance of this metabolite in mitochondria, such as mtACP and PANK2. Moreover, we measured LIP levels using a calcein assay.

**Figure 5:**
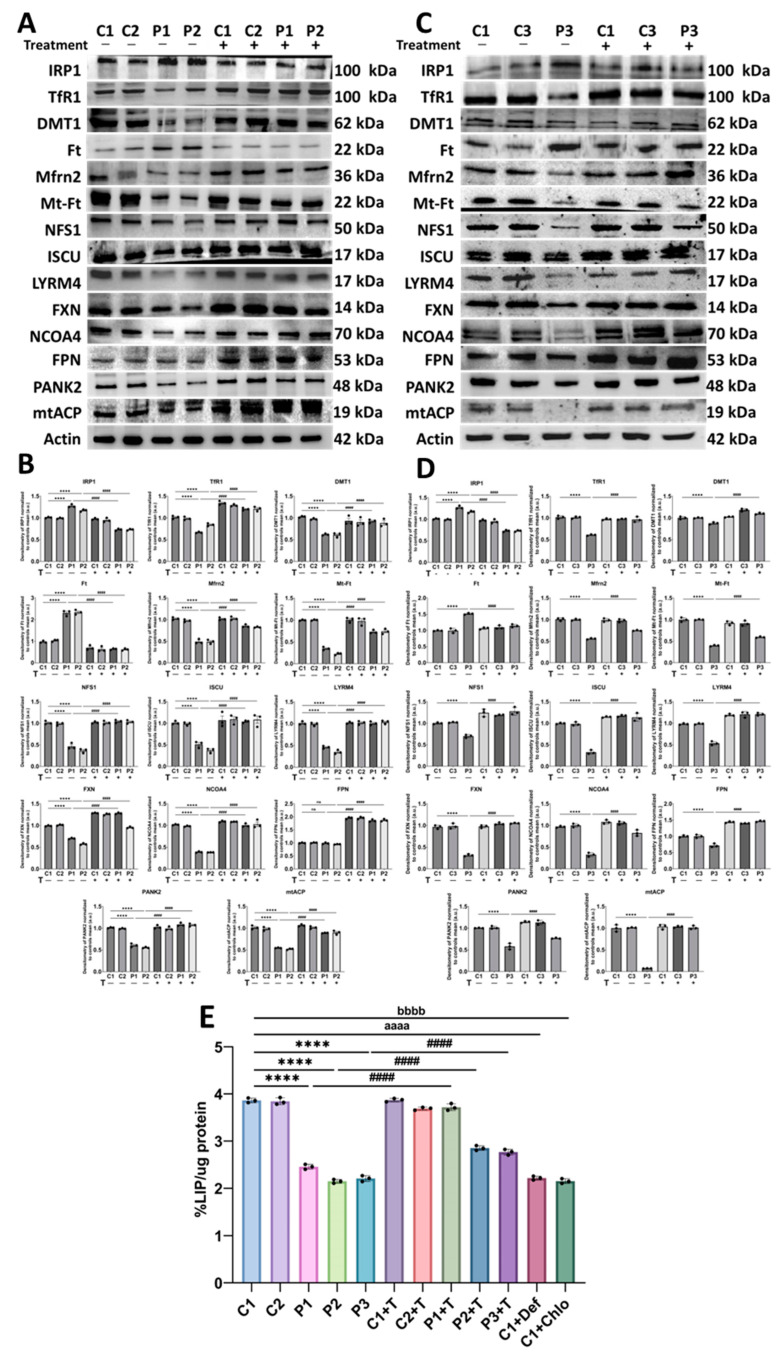
Iron metabolism in untreated and treated (+T) control and patients’ cells. Polydatin and nicotinamide were administered for seven days. (**A**) Immunoblotting analysis of iron metabolism-related proteins in control (C1 and C2) and patient (P1 and P2) cells. (**B**) Band densitometry of Western blot data, referred to actin and normalized to the mean of controls. Data represent the mean ± SD of three independent experiments. Number of biological replicates = 3. **** *p* < 0.0001 between control and mutant fibroblasts. ^####^ *p* < 0.0001 between untreated and treated patients’ cells (**C**) Immunoblotting analysis of iron metabolism-related proteins in control (C1 and C3) and patient (P3) cells. (**D**) Band densitometry of Western blot data, referred to actin and normalized to the mean of controls. (**E**) LIP levels determined using a calcein assay. C1 cells treated with 10 µM chloramphenicol (C1+Chlo) were used to mimic the mitochondrial protein synthesis deficiency. C1 cells treated with 100 µM deferiprone (C1+Def) served as a negative control. Data represent the mean ± SD of three independent experiments. Number of biological replicates = 3. **** *p* < 0.0001 between control and mutant fibroblasts. ^####^ *p* < 0.0001 between untreated and treated patients’ cells. ^aaaa^ *p* < 0.0001 between untreated and deferiprone-treated C1 fibroblasts. ^bbbb^ *p* < 0.0001 between untreated and chloramphenicol-treated C1 cells. a.u.: arbitrary units. Refer to Supplementary Figure S6 for comparison between grouped data of controls and patients.

Interestingly, we observed altered expression levels of all analyzed proteins in mutant fibroblasts compared to control cells, which were efficiently restored after supplementation with polydatin and nicotinamide (Figure 5A–D). LIP levels were significantly lower in patients’ fibroblasts than in control cells, suggesting impaired iron handling. Notably, treatment with polydatin and nicotinamide increased LIP levels to those observed in control cells. Control cells treated with 100 µM deferiprone were used as a negative control. Additionally, control cells treated with 10 µM chloramphenicol to simulate the mitochondrial protein synthesis deficiency of mutant fibroblasts exhibited lower LIP levels compared to untreated control fibroblasts, suggesting that the impaired iron management in patient-derived cells is associated with mitochondrial dysfunction (Figure 5E).

**Figure 6:**
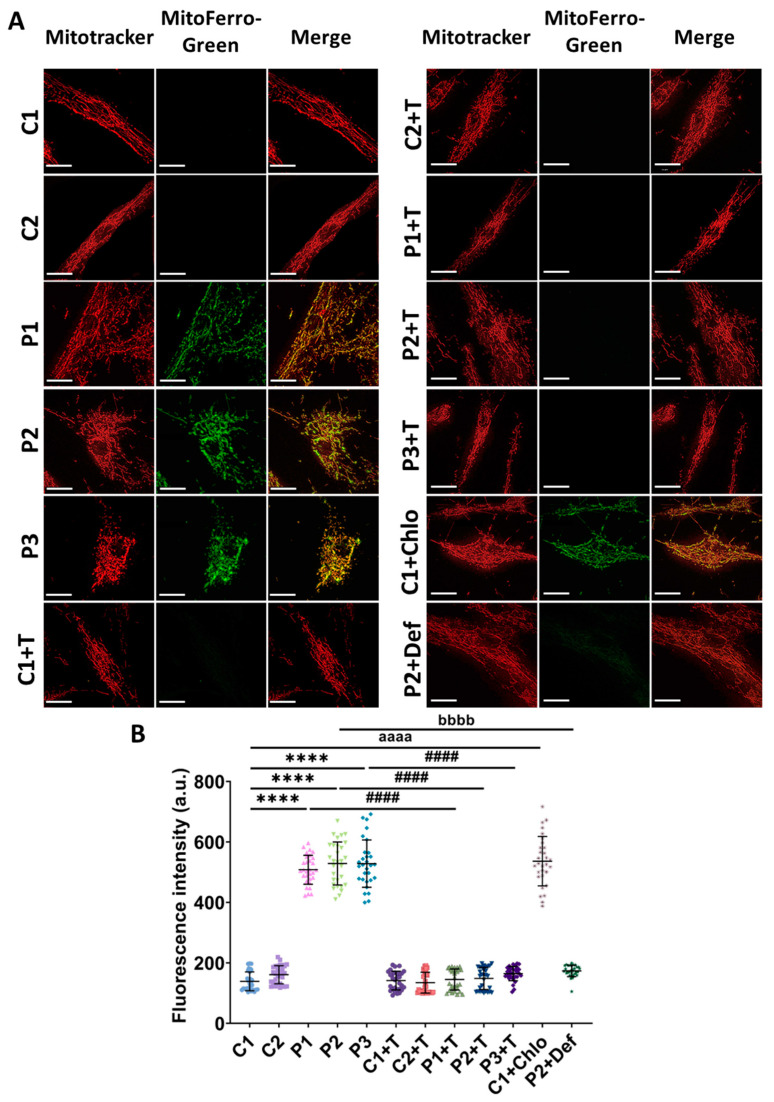
Mitochondrial iron levels in untreated and treated (+T) control (C1 and C2) and patient (P1, P2, and P3) cells. Polydatin and nicotinamide were used for seven days. P2 cells treated with 100 µM deferiprone (P2+Def) were used as a negative control. C1 cells treated with 10 µM chloramphenicol (C1+Chlo) were used to simulate mitochondrial protein synthesis deficiency. (**A**) Representative images of mitochondrial iron detected using Mito-FerroGreen. Cells were also stained with Mitotracker^TM^ Deep Red to visualize the colocalization of both probes. Scale bar: 20 µm. (**B**) Quantification of Mito-FerroGreen fluorescence intensity. Data represent the mean ± SD of three independent experiments (at least 30 images were taken from each condition and experiment). Number of biological replicates = 3. **** *p* < 0.0001 between control and mutant fibroblasts. ^####^ *p* < 0.0001 between untreated and treated patients’ cells. ^aaaa^ *p* < 0.0001 between untreated and chloramphenicol-treated C1 fibroblasts. ^bbbb^ *p* < 0.0001 between untreated and deferiprone-treated P2 cells. a.u.: arbitrary units. Refer to Supplementary Figure S7 for comparison between grouped data of controls and patients.

Given the observed alterations in iron metabolism and the formation of lipofuscin-like granules within mitochondria, we next used Mito-FerroGreen to detect mitochondrial iron levels. Cells from patients displayed higher fluorescence intensity compared to control fibroblasts, indicating increased iron accumulation within these organelles. A similar increase was observed when C1 cells were treated with 10 µM chloramphenicol. Remarkably, polydatin and nicotinamide supplementation significantly reduced mitochondrial iron accumulation in mutant fibroblasts. As a negative control, P2 cells were treated with 100 µM deferiprone (Figure 6).

### 3.4. Polydatin and Nicotinamide Correct Lipid Peroxidation in Mutant GFM1 Fibroblasts

**Figure 7:**
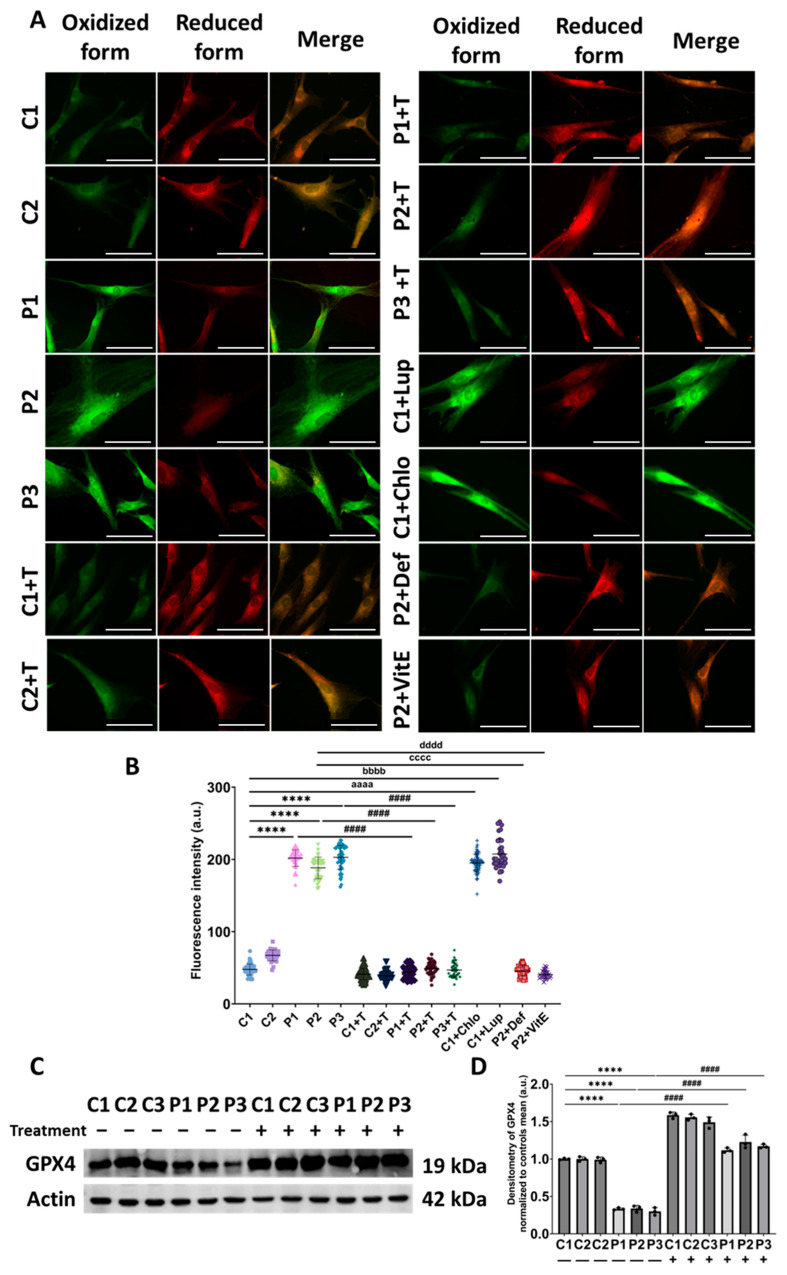
Lipid peroxidation in untreated and treated (+T) control (C1 and C2) and patient (P1, P2, and P3) cells. Polydatin and nicotinamide were administered for seven days. C1 cells were treated with 10 µM chloramphenicol (C1+Chlo) to mimic the mitochondrial protein synthesis deficiency. C1 cells treated with 500 µM Luperox^®^ (C1+Lup) served as a positive control for induced lipid peroxidation. P2 cells were treated with 100 µM deferiprone (P2+Def) to confirm the dependence of lipid peroxidation on iron. P2 cells were treated with 50 µM Vitamin E (P2+VitE) as a negative control. (**A**) Representative images of lipid peroxidation using BODIPY^®^ 581/591 C11 staining. Scale bar: 20 µm. (**B**) Quantification of oxidized fluorescence intensity. (**C**) Immunoblotting analysis of GPX4 in control (C1, C2, and C3) and patient (P1, P2, and P3) cells. (**D**) Band densitometry of Western blot data, referred to actin and normalized to the mean of controls. Data represent the mean ± SD of three independent experiments (at least 30 images were taken for each condition and experiment). **** *p* < 0.0001 between control and mutant fibroblasts. Number of biological replicates = 3. ^####^ *p* < 0.0001 between untreated and treated patients’ cells. ^aaaa^ *p* < 0.0001 between untreated and chloramphenicol-treated C1 fibroblasts. ^bbbb^ *p* < 0.0001 between untreated and Luperox^®^-treated C1 cells. ^cccc^ *p* < 0.0001 between untreated and deferiprone-treated P2 cells. ^dddd^ *p* < 0.0001 between untreated and Vitamin E-treated P2 cells. a.u.: arbitrary units. Refer to Supplementary Figure S8 for comparison between grouped data of controls and patients.

Given that iron accumulation and lipid peroxidation establish a vicious cycle [13], we next assessed cellular lipid peroxidation in mutant *GFM1* fibroblasts using Bodipy^®^ 581/591 C11. We observed that the levels of cellular lipid peroxidation were significantly higher in patients’ fibroblasts compared to control cells. Notably, treatment with polydatin and nicotinamide significantly mitigated this pathological feature in patients’ fibroblasts. To model the mitochondrial protein synthesis deficiency characteristic of patients’ fibroblasts, control cells were treated with 10 µM chloramphenicol. Additionally, lipid peroxidation was induced in control cells by treating them with 500 µM Luperox^®^. Both chloramphenicol and Luperox^®^ treatments increased fluorescence intensity, indicating elevated lipid peroxidation. To evaluate potential antioxidant effects, P2 cells were treated with 50 µM vitamin E, a well-known antioxidant, and 100 µM deferiprone, an iron chelator. Supplementation with these compounds significantly reduced fluorescence intensity, suggesting that lipid peroxidation is dependent on iron and oxidative stress (Figure 7A,B).

Furthermore, as GPX4, the primary enzyme responsible for neutralizing membrane lipid hydroperoxides, plays a central role in mitigating lipid peroxidation, we performed Western blot analysis to evaluate GPX4 expression levels. Patients’ fibroblasts exhibited significantly lower GPX4 expression levels compared to control cells. Remarkably, supplementation with polydatin and nicotinamide increased GPX4 expression in patient-derived fibroblasts, suggesting that this treatment effectively reduces lipid peroxidation (Figure 7C).

**Figure 8:**
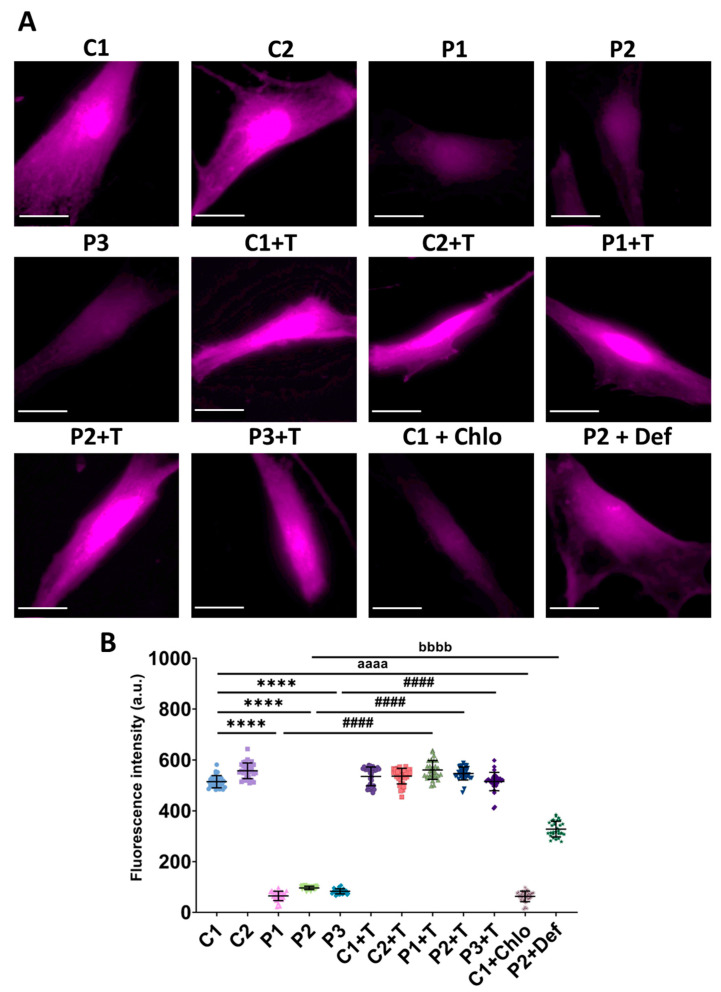
GSH levels in untreated and treated (+T) control (C1 and C2) and patient (P1, P2, and P3) cells. Polydatin and nicotinamide were administered for seven days. C1 cells were treated with 10 µM chloramphenicol (C1+Chlo) to mimic the mitochondrial protein synthesis deficiency. P2 cells were treated with 100 µM deferiprone (P2+Def) to evaluate the dependence of GSH levels on iron. (**A**) Representative images of GSH levels using ThiolTracker^TM^ Violet staining. Scale bar: 20 µm. (**B**) Quantification of fluorescence intensity. Data represent the mean ± SD of three independent experiments (at least 30 images were taken for each condition and experiment). Number of biological replicates = 3. **** *p* < 0.0001 between control and mutant fibroblasts. ^####^ *p* < 0.0001 between untreated and treated patients’ cells. ^aaaa^ *p* < 0.0001 between untreated and chloramphenicol-treated C1 fibroblasts. ^bbbb^ *p* < 0.0001 between untreated and deferiprone-treated P2 cells. a.u.: arbitrary units. Refer to Supplementary Figure S8 for comparison between grouped data of controls and patients.

We also evaluated GSH levels, a critical cofactor for GPX4, using ThiolTracker^TM^ Violet. Patients’ cells showed lower fluorescence intensity compared to control cells, a deficit that was significantly improved by polydatin and nicotinamide treatment. To simulate mitochondrial protein synthesis deficiency, control cells were treated with 10 µM chloramphenicol, resulting in lower fluorescence intensity, comparable to that of patients’ cells. This finding indicates that the reduction in GSH is attributable to the mitochondrial protein synthesis defect characteristic of mutant *GFM1* fibroblasts. In contrast, P2 cells treated with 100 µM deferiprone exhibited increased fluorescence intensity compared to untreated P2 cells, suggesting that reducing iron accumulation and lipid peroxidation can restore GSH levels (Figure 8).

**Figure 9:**
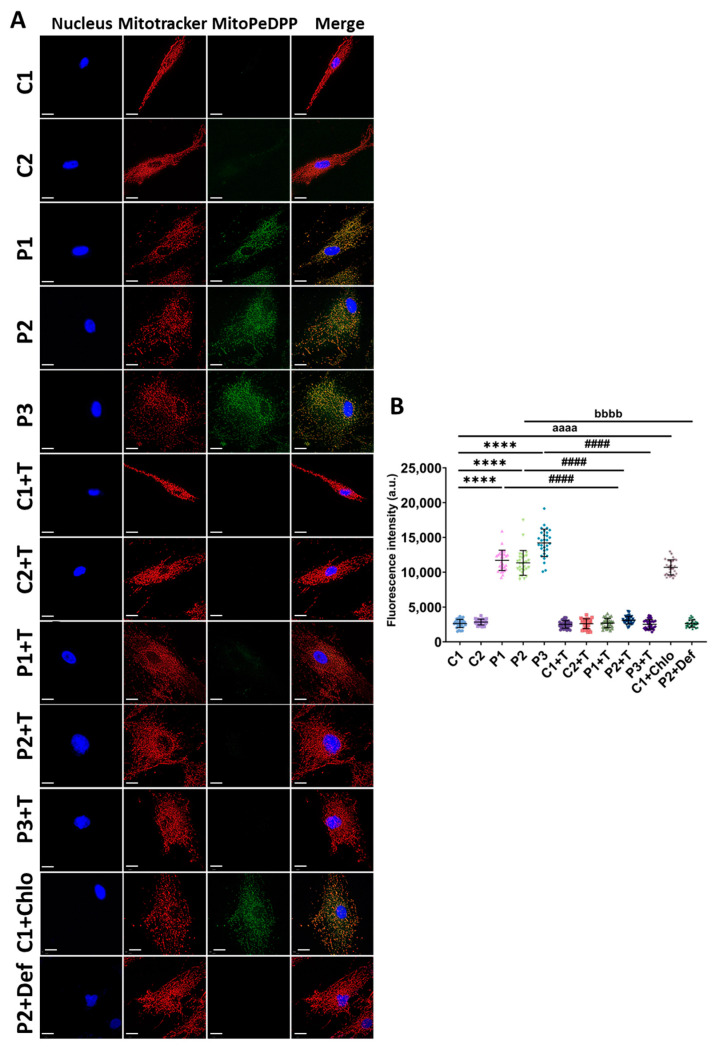
Mitochondrial lipid peroxidation in untreated and treated (+T) control (C1 and C2) and patient (P1, P2, and P3) cells. Cells were treated with polydatin and nicotinamide for seven days. C1 cells were treated with 10 µM chloramphenicol (C1+Chlo) to simulate the mitochondrial protein synthesis failure of patients’ fibroblasts. P2 cells were treated with 100 µM deferiprone (P2+Def) as a negative control. (**A**) Representative images of mitochondrial lipid peroxidation using MitoPeDPP^®^ staining. Cells were also stained with Mitotracker^TM^ Deep Red FM to visualize the mitochondrial network. Nuclei were visualized by DAPI staining. Scale bar: 20 µm. (**B**) Quantification of fluorescence intensity. Data represent the mean ± SD of three independent experiments (at least 30 images were taken for each condition and experiment). Number of biological replicates = 3. **** *p* < 0.0001 between control and mutant fibroblasts. ^####^ *p* < 0.0001 between untreated and treated patients’ cells. ^aaaa^ *p* < 0.0001 between untreated and chloramphenicol-treated C1 fibroblasts. ^bbbb^ *p* < 0.0001 between untreated and deferiprone-treated P2 cells. a.u.: arbitrary units. Refer to Supplementary Figure S8 for comparison between grouped data of controls and patients.

Moreover, mitochondrial lipid peroxidation has recently been identified as playing a critical role in inducing ferroptosis, highlighting this importance in this type of cell death [41]. To investigate this process, we measured mitochondrial lipid peroxidation in control and mutant fibroblasts using MitoPeDPP^®^. Our results showed that mitochondrial lipid peroxidation was significantly higher in patients’ fibroblasts in comparison to control cells. This pathological condition was efficiently corrected by polydatin and nicotinamide treatment. Consistent with our observations of cellular lipid peroxidation, control cells treated with 10 µM chloramphenicol displayed increased mitochondrial lipid peroxidation. In P2 cells treated with 100 µM deferiprone, fluorescence intensity was markedly lower compared to untreated P2 cells, indicating that iron chelation reduces mitochondrial lipid peroxidation (Figure 9).

### 3.5. Polydatin and Nicotinamide Prevent Erastin-Induced Ferroptosis in Mutant GFM1 Fibroblasts

**Figure 10:**
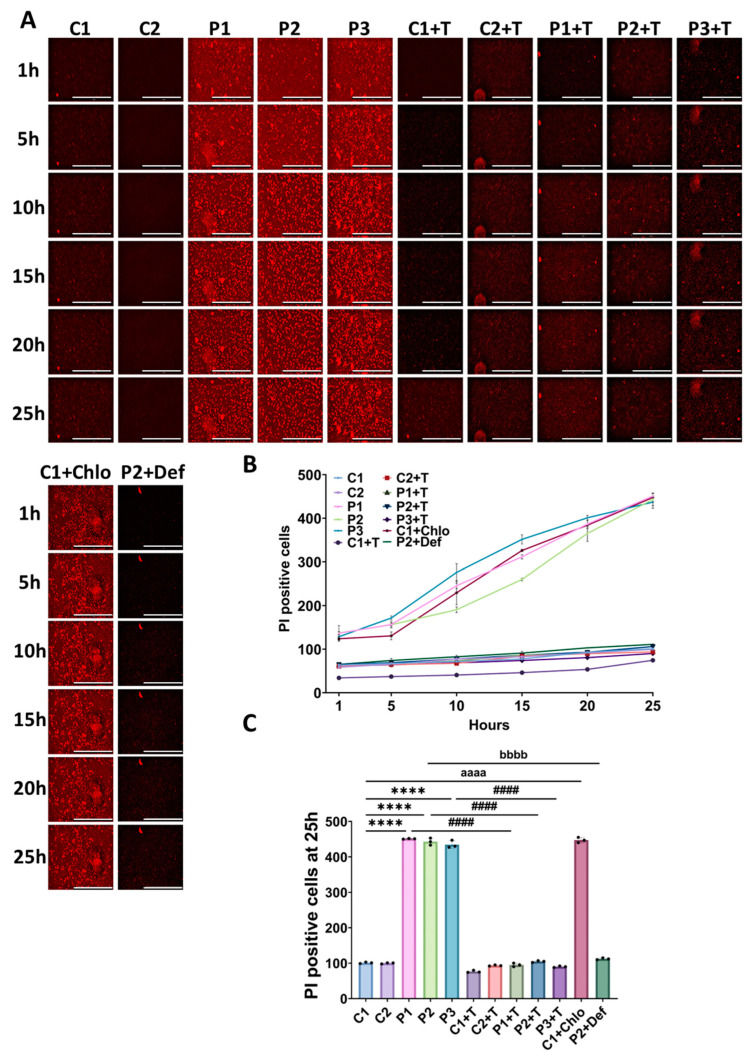
Ferroptosis sensitivity in untreated and treated (+T) control (C1 and C2) and patient (P1, P2, and P3) fibroblasts. Polydatin and nicotinamide were used for seven days. C1 cells treated with 10 µM chloramphenicol (C1+Chlo) were used to mimic the patients’ pathogenic variant. P2 cells treated with 100 µM deferiprone (P2+Def) were used as a negative control. (**A**) Representative images of dead cells staining with PI over the course of 25 h after the supplementation with 5 µM erastin. Scale bar: 20 µm. (**B**) Quantification of PI positive cells over the period of 25 h. (**C**) Quantification of PI positive cells at 25 h after the addition of erastin. Data represent the mean ± SD of three separate experiments (at least 30 images were taken for each condition and experiment). Number of biological replicates = 3. **** *p* < 0.0001 between control and mutant fibroblasts. ^####^ *p* < 0.0001 between untreated and treated patients’ cells. ^aaaa^ *p* < 0.0001 between untreated and chloramphenicol-treated C1 fibroblasts. ^bbbb^ *p* < 0.0001 between untreated and deferiprone-treated P2 cells. Refer to Supplementary Figure S9 for comparison between grouped data of controls and patients.

Next, we examined ferroptosis induced by erastin, a well-characterized ferroptosis inducer that depletes glutathione and increases lipid ROS levels [42]. Remarkably, patients’ fibroblasts showed greater susceptibility to erastin-induced ferroptosis in comparison to control cells. Supplementation with polydatin and nicotinamide significantly mitigated erastin-induced ferroptosis in patients’ fibroblasts. To model the mitochondrial protein synthesis deficiency characteristic of mutant *GFM1* fibroblasts, control cells were treated with 10 µM chloramphenicol. This treatment significantly increased susceptibility to erastin-induced cell death, further highlighting the connection between mitochondrial dysfunction and ferroptosis. Moreover, P2 cells were treated with 100 µM deferiprone as a negative control, which resulted in diminished susceptibility to erastin-induced ferroptosis (Figure 10).

### 3.6. Polydatin and Nicotinamide Reduce Intracellular Iron Accumulation in Mutant GFM1 Induced Neurons

In mitochondrial diseases, the nervous and muscular systems are predominantly affected due to their high energy demands. Although fibroblasts provide valuable cellular models for these diseases, we reprogrammed P2 cells into induced neurons (iNs) via direct reprogramming to further elucidate the beneficial effects of polydatin and nicotinamide supplementation.

Thirty days post-infection, the cells demonstrated immunoreactivity against Tau, a microtubule-associated protein localized in neuronal axons. Based on Tau^+^ cells, we determined conversion efficiency and neuronal purity. In control cells, the conversion efficiency was 20.05 ± 3.74%, while in mutant cells, it was 19.36 ± 2.84%. Neuronal purity was 69.51 ± 5.24% in control cells and 68.25 ± 5.41% in mutant cells.

**Figure 11:**
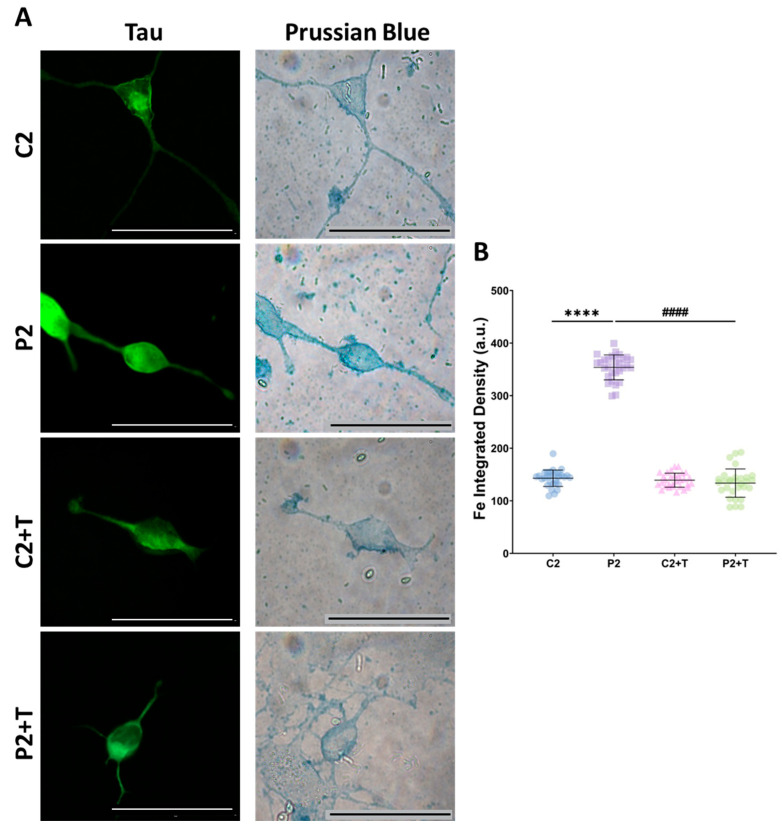
Intracellular iron accumulation in untreated and treated (+T) control (C2) and patient (P2) iNs. Polydatin and nicotinamide treatment was applied for seven days. (**A**) Representative images of Prussian Blue staining and Tau immunofluorescence. Scale bar: 20 µm. (**B**) Quantification of iron integrated density. Data represent the mean ± SD of three independent experiments (at least 30 images were taken for each condition and experiment). Number of biological replicates = 3. **** *p* < 0.0001 between control and mutant fibroblasts. ^####^ *p* < 0.0001 between untreated and treated patients’ cells. a.u.: arbitrary units.

As expected, mutant iNs exhibited significantly higher intracellular iron accumulation, as determined by Prussian Blue staining, compared to control iNs. Notably, polydatin and nicotinamide treatment reduced iron accumulation in P2 iNs to levels comparable to those observed in control iNs (Figure 11).

**Figure 12:**
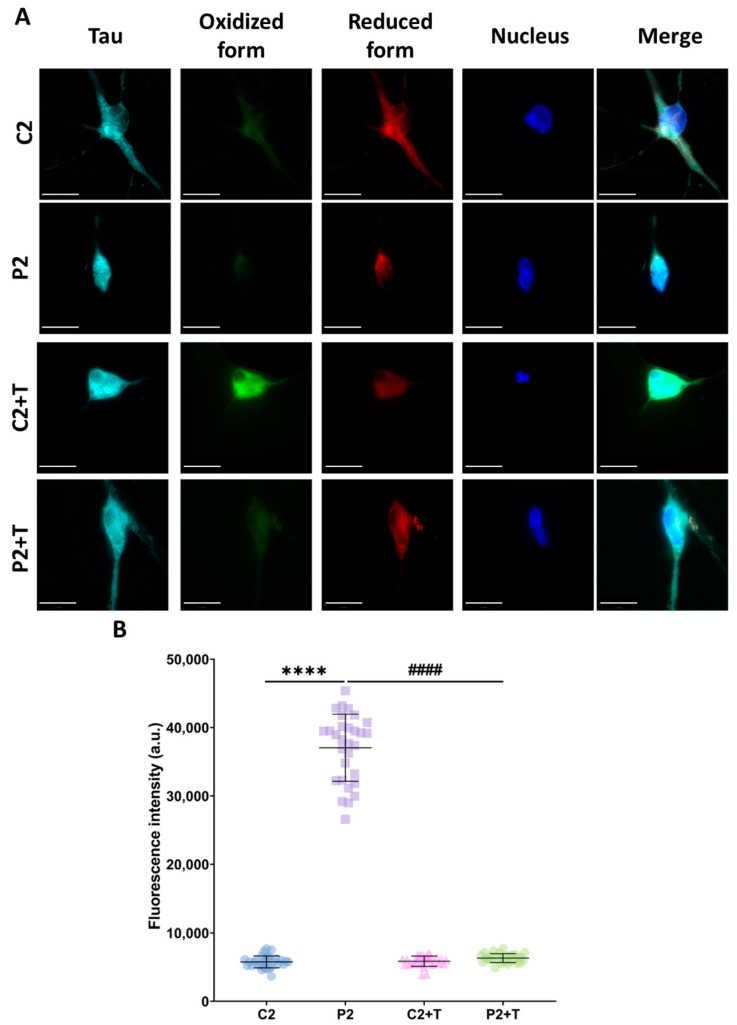
Lipid peroxidation in untreated and treated (+T) control (C2) and patient (P2) iNs. Polydatin and nicotinamide were administered for seven days. (**A**) Representative images of lipid peroxidation using BODIPY^®^ 581/591 C11 staining. Scale bar: 20 µm. (**B**) Quantification of oxidized form fluorescence intensity. Data represent the mean ± SD of three independent experiments (at least 30 images were taken for each condition and experiment). Number of biological replicates = 3. **** *p* < 0.0001 between control and mutant fibroblasts. ^####^ *p* < 0.0001 between untreated and treated patients’ cells. a.u.: arbitrary units.

Additionally, we assessed cellular lipid peroxidation in C2 and P2 iNs using BODIPY^®^ 581/591 C11. Patient-derived iNs exhibited significantly higher levels of lipid peroxidation in comparison to control cells. Notably, treatment with polydatin and nicotinamide effectively reduced lipid peroxidation in P2 iNs to levels comparable to those observed in control cells (Figure 12).

### 3.7. Pathological Alterations of Mutant Fibroblasts Are Present in Two Additional Patient-Derived Cell Death Models

**Figure 13:**
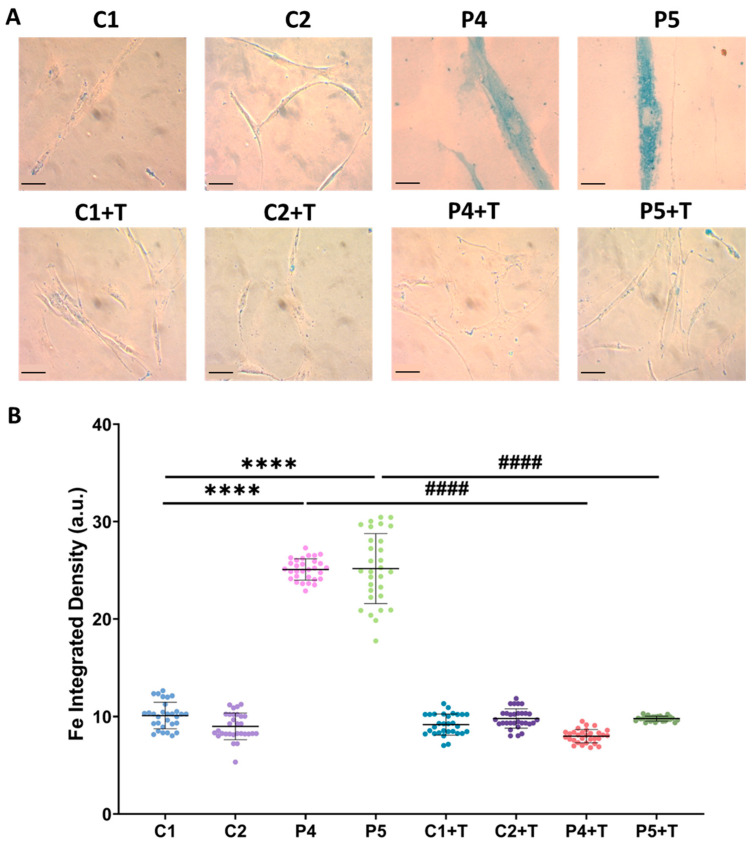
Intracellular iron accumulation in untreated and treated (+T) control (C1 and C2) and patient (P4 and P5) cells. Cells were treated with polydatin and nicotinamide for seven days. (**A**) Representative images of Prussian Blue staining. Scale bar: 20 µm. (**B**) Quantification of iron integrated density. Data represent the mean ± SD of three independent experiments (at least 30 images were taken from each condition and experiment). Number of biological replicates = 3. **** *p* < 0.0001 between control and mutant fibroblasts. ^####^ *p* < 0.0001 between untreated and treated patients’ fibroblasts. a.u.: arbitrary units.

**Figure 14:**
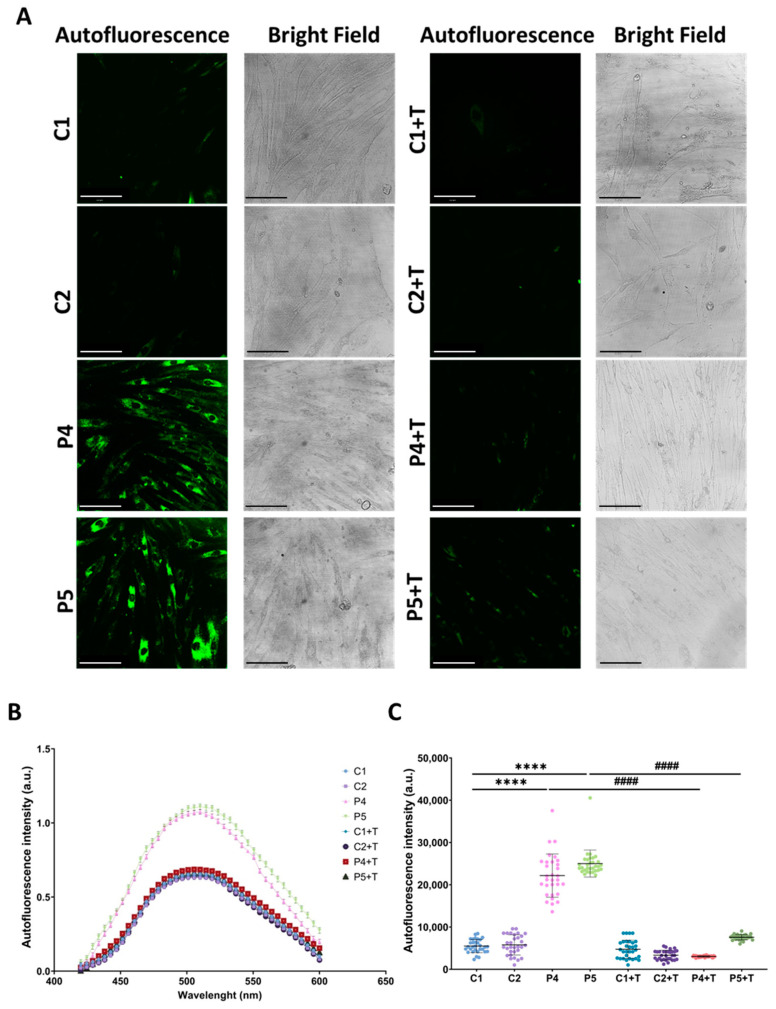
Autofluorescence analysis of untreated and treated (+T) control (C1 and C2) and patient (P4 and P5) cells. Cells were treated with polydatin and nicotinamide for seven days. (**A**) Representative images showing GFP and bright field channels of control and mutant *GFM1* fibroblasts. Scale bar: 100 µm. (**B**) Autofluorescence spectra of lipofuscin granules with excitation laser at 405 nm. (**C**) Quantification of autofluorescence intensity. Data represent the mean ± SD of three independent experiments (at least 30 images were taken from each condition and experiment). Number of biological replicates = 3. **** *p* < 0.0001 between control and mutant fibroblasts. ^####^ *p* < 0.0001 between untreated and treated patients’ cells. a.u.: arbitrary units.

**Figure 15:**
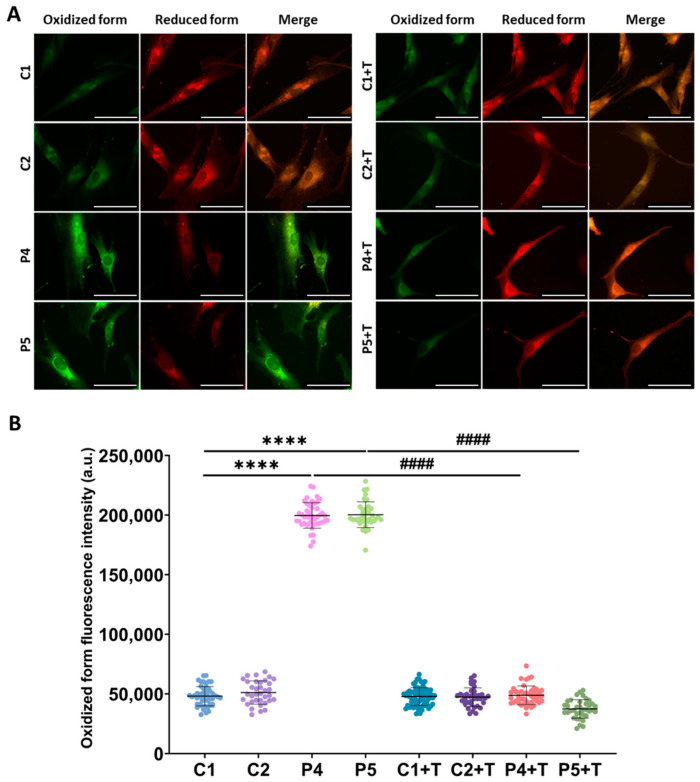
Lipid peroxidation in untreated and treated (+T) control (C1 and C2) and patient (P4 and P5) cells. Polydatin and nicotinamide were administered for seven days (+T). (**A**) Representative images of lipid peroxidation using BODIPY^®^ 581/591 C11 staining. Scale bar: 20 µm. (**B**) Quantification of oxidized fluorescence intensity. Data represent the mean ± SD of three independent experiments (at least 30 images were taken for each condition and experiment). Number of biological replicates = 3. **** *p* < 0.0001 between control and mutant fibroblasts. ^####^ *p* < 0.0001 between untreated and treated patients’ cells. a.u.: arbitrary units.

**Figure 16:**
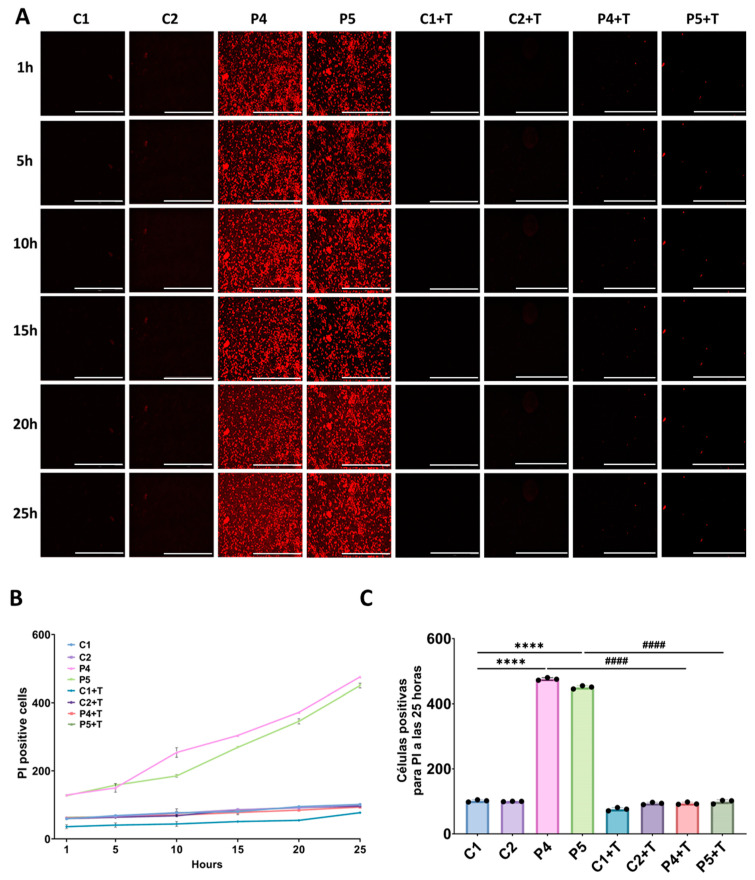
Ferroptosis sensitivity in untreated and treated (+T) control (C1 and C2) and patient (P4 and P5) fibroblasts. Polydatin and nicotinamide were used for seven days. (**A**) Representative images of dead cell staining with PI over the course of 25 h after supplementation with 5 µM erastin. Scale bar: 20 µm. (**B**) Quantification of PI positive cells over the period of 25 h. (**C**) Quantification of PI positive cells at 25 h after the addition of erastin. Data represent the mean ± SD of three separate experiments (at least 30 images were taken for each condition and experiment). Number of biological replicates = 3. **** *p* < 0.0001 between control and mutant fibroblasts. ^####^ *p* < 0.0001 between untreated and treated patients’ cells.

The observed pathological features were confirmed in fibroblasts derived from two additional patients with pathogenic *GFM1* variants (P4, P5). These mutant fibroblasts exhibited increased intracellular iron accumulation, as determined by Prussian Blue staining (Figure 13), elevated autofluorescence intensity indicative of lipofuscin-like material accumulation (Figure 14), increased lipid peroxidation, detected by Bodipy^®^ 581/591 C11 (Figure 15), and heightened sensitivity to erastin-induced ferroptosis (Figure 16). Interestingly, treatment with polydatin and nicotinamide effectively reversed all these pathological features in mutant fibroblasts, restoring them to levels comparable to those of control cells.

### 3.8. Vitamin E Reduces the Minimum Effective Concentration of Polydatin and Nicotinamide Treatment

Next, as vitamin E is known to suppress lipid peroxidation and, consequently, ferroptosis, we tested whether its supplementation could lower the effective concentrations of polydatin and nicotinamide required for treatment [43].

**Figure 17:**
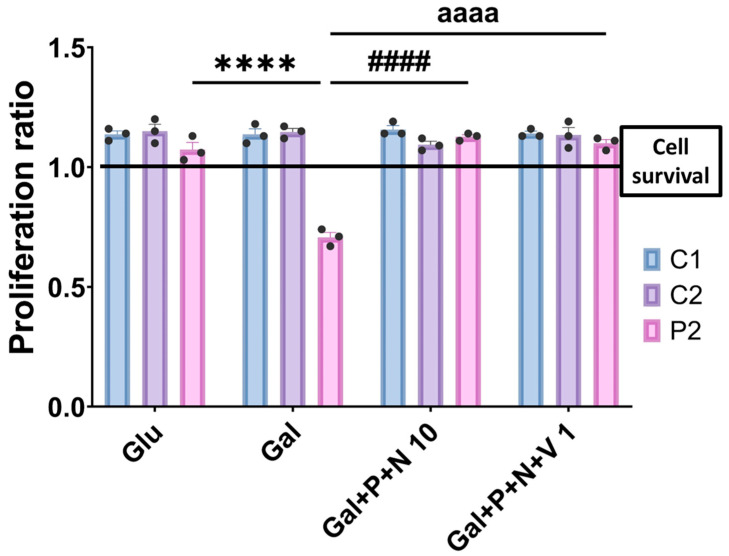
Proliferation ratio quantification of galactose screening. Control (C1 and C2) and patient (P2) fibroblasts were initially seeded in glucose medium and treated with polydatin (P) and nicotinamide (N) at 10 µM, or the combination of P, N, and vitamin E (V) at 1 µM for three days. After that, the medium was changed to galactose medium, and treatments were replenished. Cell counting was obtained immediately in that moment (considered T0) and 72 h later (considered T72) using the BioTek Cytation 1 Cell Imaging Multi-Mode Reader. The proliferation ratio was calculated by dividing the number of cells at T72 by the number of cells at T0. Results equal to 1 denote cell survival, values below 1 indicate cell death, and values greater than 1 denote cell proliferation. Data represent the mean ± SD of three independent experiments. Number of biological replicates = 3. **** *p* < 0.0001 between patient cells in glucose and galactose medium. ^####^ *p* < 0.0001 between patient fibroblasts in galactose medium and galactose medium plus polydatin and nicotinamide at 10 µM. ^aaaa^ *p* < 0.0001 between patient cells in galactose medium and galactose medium plus polydatin, nicotinamide, and vitamin E at 1 µM. Refer to Supplementary Figure S10 for representative images of the galactose screening assay. Glu: glucose. Gal: galactose.

We cultured cells in galactose medium, a nutrient-restrictive condition in which patients’ cells undergo cell death due to their mitochondrial dysfunction. In a previous study by our laboratory, fibroblasts derived from patients with pathogenic *GFM1* variants were treated with 10 µM polydatin and 10 µM nicotinamide, establishing these concentrations as the minimum effective doses [24]. In the current study, we treated cells with 1 µM each of polydatin, nicotinamide, and vitamin E. This combination allowed for the survival of mutant cells in galactose medium without inducing significant changes in control cells (Figure 17).

These findings suggest that vitamin E supplementation may lower the minimum concentrations of polydatin and nicotinamide required for effective treatment in patients with pathogenic *GFM1* variants.

**Figure 18:**
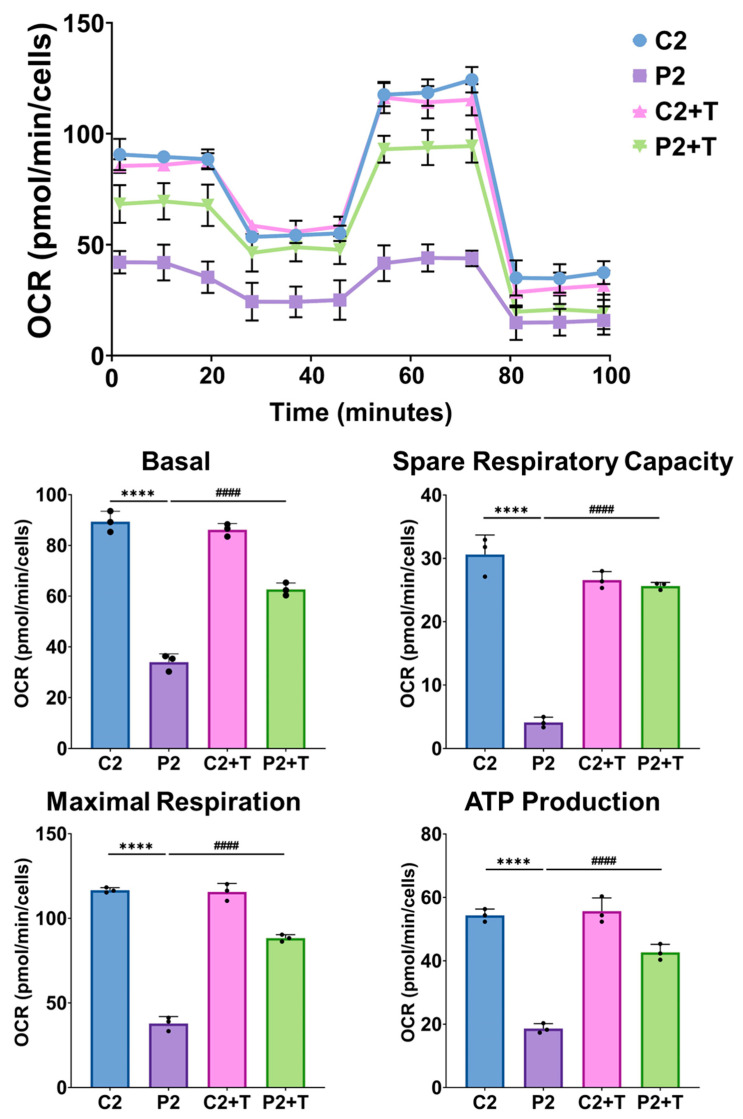
Effect of treatment with polydatin, nicotinamide, and vitamin E at 1 µM on the Mitostress bioenergetic assay in untreated and treated (+T) control (C2) and patient (P2) fibroblasts. The mitochondrial respiration profile was measured utilizing the SeaHorse XFe24 flux analyzer. Cells were treated with polydatin, nicotinamide, and vitamin E over seven days. Data represent the mean ± SD of three separate experiments. Number of biological replicates = 3. **** *p* < 0.0001 between control and patient fibroblasts. ^####^ *p* < 0.0001 between untreated and treated P2 cells. OCR: oxygen consumption rate.

**Figure 19:**
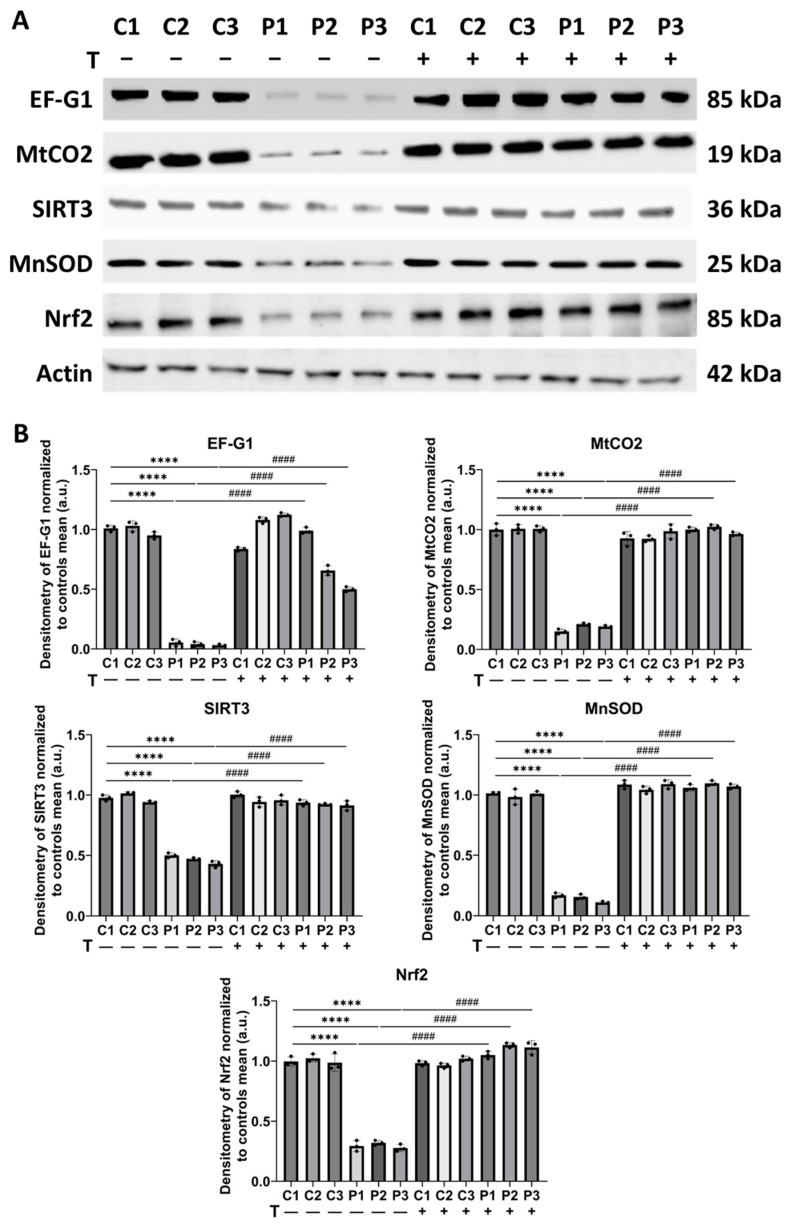
Effect of treatment with polydatin, nicotinamide, and vitamin E at 1 µM (T) on the protein expression levels of EF-G1, MtCO2, SIRT3, MnSOD, and Nrf2 in control and patient fibroblasts. Polydatin, nicotinamide, and vitamin E were administered for seven days. (**A**) Immunoblotting analysis in control (C1, C2, and C3) and patient (P1, P2, and P3) cells. (**B**) Band densitometry of Western blot data, referred to actin and normalized to the mean of controls. Number of biological replicates = 3. **** *p* < 0.0001 between control and mutant fibroblasts. ^####^ *p* < 0.0001 between untreated and treated patients’ cells. a.u.: arbitrary units.

To confirm the synergistic effect of vitamin E, we performed a Mitostress test assay using an XFe24 extracellular flux analyzer (SeaHorse Bioscience, Billerica, MA, USA) in C2 and P2 cells to evaluate the impact of the treatment on the bioenergetic profile. Mutant P2 fibroblasts showed a significant reduction in all analyzed parameters—basal respiration, maximal respiration, spare respiratory capacity, and ATP production—in comparison to C2 cells. Notably, combined treatment with polydatin, nicotinamide, and vitamin E at 1 µM significantly improved these bioenergetic parameters in P2 fibroblasts (Figure 18). Additionally, to further confirm the therapeutic effects of the polydatin, nicotinamide, and vitamin E combination, we analyzed the protein expression levels of key proteins. This analysis included: the mutant protein EF-G1, the mitochondrially encoded cytochrome C oxidase subunit II (MtCO2), and three proteins involved in the antioxidant response, such as SIRT3, the target of our previous treatment, MnSOD, a key mitochondrial antioxidant protein, and nuclear respiratory factor 2 (Nrf2), a master regulator of cellular antioxidant response. We observed a significant reduction in the expression levels of all analyzed proteins in patient-derived fibroblasts compared to control cells. However, supplementation of polydatin, nicotinamide, and vitamin E notably increased the protein expression levels of all analyzed proteins (Figure 19).

## 4. Discussion

For most mitochondrial diseases, there are currently no curative therapeutic options [22]. Existing approaches primarily focus on symptomatic management or specific interventions for certain disorders. For example, idebenone is used for patients with Leber’s Hereditary Optic Neuropathy (LHON) [44,45], while thiamine, riboflavin, biotin, or niacin are employed to address cofactor deficiency mitochondrial disorders [46]. Additionally, authorized treatments, such as taurine and L-arginine, are used to reduce stroke-like events in MELAS syndrome [47,48,49]. Other strategies aim to increase mitochondrial content in cells using drugs such as omaveloxone [50], bezafibrate [51,52], or acipimox [53]. However, the safety and efficacy of these treatments remain uncertain. In addition, gene therapy has emerged as a promising avenue, offering potential cures for mitochondrial diseases. Yet, to date, only one gene therapy is undergoing clinical trials specifically targeting LHON [54]. Therefore, this approach has not yet become part of routine clinical practice.

Despite these challenges, advancements in next-generation sequencing techniques have led to the increased identification of mitochondrial disease cases. This growing patient population underscores the urgent need to further investigate mitochondrial diseases and develop innovative therapeutic strategies, which is the primary focus of this study.

The study of ferroptosis has rapidly expanded in recent years, particularly due to its implications in cancer therapies, ischemic injuries, and neurodegeneration [55,56,57,58,59,60,61,62,63]. Ferroptosis is a distinct form of non-apoptotic cell death characterized by the accumulation of iron and the excessive peroxidation of lipids [64].

Firstly, our study demonstrates that mitochondrial dysfunction is linked to ROS overproduction, as fibroblasts derived from patients with pathogenic *GFM1* variants exhibited elevated superoxide anion levels, indicating a state of oxidative stress. Interestingly, polydatin and nicotinamide treatment reduced superoxide anion levels in mutant fibroblasts, restoring them to levels comparable to those of control cells.

We further explored iron accumulation, which is a common pathological feature across various diseases, particularly neurodegenerative disorders such as Alzheimer’s Disease [65], Parkinson’s Disease [66], Huntington’s Disease [67], Friedreich’s Ataxia [68], and Multiple Sclerosis [69]. Iron accumulation is also a defining characteristic of a group of genetic disorders collectively known as Neurodegeneration with Brain Iron Accumulation (NBIA) [70]. Mitochondrial dysfunction is closely linked to these conditions and is associated with iron dysregulation [31,32,38]. Mitochondria play a crucial role in cellular iron homeostasis, as they are the sites for iron–sulfur clustering and heme biosynthesis, which are both essential for cellular function. Thus, mitochondrial dysfunction can lead to abnormal iron accumulation and disrupt overall cellular homeostasis. Fibroblasts derived from patients with pathogenic *GFM1* variants exhibited iron accumulation, altered the expression of iron metabolism-related proteins, and reduced levels of LIP compared to control cells. These findings suggest that mitochondrial dysfunction impairs iron management within mitochondria, causing iron buildup. Treatment with polydatin and nicotinamide effectively reduced this iron accumulation, restored the expression of iron metabolism-related proteins, and increased LIP levels, thereby reversing the pathological features observed in patient-derived fibroblasts.

Interestingly, we also observed that the accumulated iron was present in the form of lipofuscin, as detected by autofluorescence and electron microscopy. This lipofuscin accumulation resembled that seen in cellular and animal models of other pathologies, such as Pantothenate Kinase-Associated Neurodegeneration (PKAN) [31], PLA2G6-Associated Degeneration (PLAN) [32], β-propeller Protein-Associated Neurodegeneration (BPAN) [38], and retinal degeneration [71]. Electron microscopy revealed that lipofuscin-like granules formed inside mitochondria and then released to the cytoplasm, likely as a protective mechanism to remove damaged components. Remarkably, treatment with polydatin and nicotinamide reduced the number of lipofuscin-like granules in *GFM1* fibroblasts, restoring levels to those observed in control cells.

Iron accumulation is known to induce lipid peroxidation, creating a vicious cycle that exacerbates cellular injury [13]. In fibroblasts derived from patients, we observed a significant increase in oxidized lipid fluorescence in both cellular and mitochondrial membranes in comparison to control fibroblasts. Treatment with polydatin and nicotinamide corrected this lipid peroxidation in mutant fibroblasts. Additionally, chloramphenicol treatment of control cells induced lipid peroxidation, suggesting that the mitochondrial protein synthesis deficiency in patients’ fibroblasts is responsible for the elevated lipid peroxidation. Furthermore, Western blot analysis of GPX4, a key enzyme in neutralizing lipid peroxides, revealed significantly lower protein expression levels in patients’ cells compared to control fibroblasts, indicating an impaired antioxidant system. GSH levels were also reduced in mutant fibroblasts. This is consistent with observations in neurodegenerative diseases, in which the antioxidant system is often depleted or down-regulated, leading to increased oxidative stress, lipid peroxidation, and overall cellular dysfunction [72]. Notably, treatment with polydatin and nicotinamide significantly increased GPX4 expression levels and GSH in mutant fibroblasts. This effect is likely related to the activation of the mtUPR, the target of our treatment, as demonstrated in a previous publication [24]. In that study, we showed that the combination of polydatin and nicotinamide activated the mtUPR, with SIRT3 as its target, since the inhibition of SIRT3 prevented the treatment from being effective. The rationale behind using nicotinamide in our treatment lies in the fact that nicotinamide is a precursor of NAD^+^, which is a crucial cofactor for mitochondrial function and for sirtuins [73]. In fact, we have demonstrated that the combination of polydatin and nicotinamide increased NAD^+^ levels and enhanced SIRT3 deacetylase activity, leading to the activation of the mitochondrial antioxidant response [24], as has been reviewed in [74]. This, in turn, induced the expression of proteins such as manganese superoxide dismutase (MnSOD) and nuclear respiratory factor 2 (Nrf2) [75]. Nrf2 has been reported to regulate ferroptosis by reducing lipid peroxidation and increasing GPX4 protein levels, ultimately protecting cells from death [76,77].

Having established the presence of two key hallmarks of ferroptosis, iron accumulation and lipid peroxidation, as well as reduced GSH levels, we assessed ferroptosis sensitivity. We used erastin, a well-known ferroptosis inducer, to evaluate the susceptibility of mutant *GFM1* fibroblasts to ferroptosis [78]. Our results showed that fibroblasts derived from patients exhibited elevated cell death following erastin treatment in comparison to control cells. As expected, treatment with polydatin and nicotinamide reduced cell death to levels comparable to control cells, further confirming the protective effects of these compounds in mitigating oxidative stress.

In this study, we also validated the three primary pathological features—iron accumulation, lipid peroxidation, and increased sensitivity to ferroptosis—in fibroblasts derived from two additional patients and confirmed the beneficial effect of polydatin and nicotinamide treatment.

Furthermore, recognizing that neurons are among the most affected cells in mitochondrial diseases, we reprogrammed fibroblasts derived from the control and patient P2 into iNs via direct reprogramming. These iNs, which were confirmed via Tau immunoreactivity, exhibited increased intracellular iron accumulation and increased lipid peroxidation, both of which were corrected by polydatin and nicotinamide supplementation.

Finally, considering the oxidative stress environment and increased lipid peroxidation, we hypothesized that a combined treatment consisting of polydatin, nicotinamide, and vitamin E, a membrane antioxidant, might be more effective. For that reason, we conducted a galactose drug screening with 1 µM of each compound, observing that this treatment allowed the survival of mutant cells in a galactose medium, suggesting a synergistic effect. This finding is particularly relevant for clinical practice, as 1 µM is a concentration that can be more easily achieved in blood and brain [79,80], potentially enhancing the efficacy of polydatin and nicotinamide treatment for mitochondrial patients with *GFM1* pathological variants. It is important to consider that the three mentioned compounds have been reported to cross the blood–brain barrier [81,82,83], a characteristic that is crucial for targeting neuronal damage in mitochondrial patients. However, it is important to note that although the addition of vitamin E reduced the minimum effective concentration of polydatin and nicotinamide, additional studies are needed to determine whether these effects can be generalized to other cellular parameters.

In summary, mitochondrial dysfunction caused by pathological variants in the *GFM1* gene leads to ROS overproduction and improper mitochondrial iron management, resulting in iron accumulation within mitochondria. This excess iron can react with ROS via the Fenton reaction, generating more ROS and exacerbating oxidative damage. These ROS can damage proteins, DNA/RNA, and lipids, leading to lipid peroxidation. Additionally, oxidative damage to transcription factors can impair their function, causing a generalized reduction in gene transcription [84]. These detrimental effects, resulting from mitochondrial dysfunction, may ultimately lead to cell death by ferroptosis. However, treatment with polydatin and nicotinamide halts these pathological processes in mutant fibroblasts, making it a promising therapeutic approach for mitochondrial patients. Moreover, the combined treatment with polydatin, nicotinamide, and vitamin E at a concentration of 1 µM allowed the survival of mutant cells in a nutrient-restrictive medium. As previously reported, treatments that support cell survival in such conditions can reverse cellular pathophysiology, suggesting that this combined therapy could also be a potential treatment for mitochondrial diseases. Nevertheless, further research is necessary, as the number of patients suffering from pathogenic *GFM1* variants is low due to the rare nature of the disease. Additionally, it is important to note that this study was conducted in cell models, and its findings may not directly translate to a whole organism. This limitation could be overcome by employing animal models, such as those described by Molina-Berenguer et al. [85], to validate these findings and explore the translational potential of this therapeutic approach.

## 5. Conclusions

Treatment with polydatin and nicotinamide demonstrated remarkable therapeutic effects, reversing major pathological markers in patients’ fibroblasts, including iron/lipofuscin accumulation, lipid peroxidation, and increased sensitivity to ferroptosis. Moreover, the addition of vitamin E improved cell survival under nutrient-restrictive conditions, suggesting a potential synergistic effect among the three compounds.

In conclusion, our findings underscore the critical role of mitochondrial dysfunction in ferroptosis and suggest that a therapeutic approach combining polydatin, nicotinamide, and vitamin E may offer a promising strategy for treating mitochondrial diseases associated with pathogenic *GFM1* variants, although confirmation in animal models is needed.

## Data Availability

Data supporting the findings of this study are not openly available due to reasons of sensitivity and to protect the privacy of individuals; however, data are available from the corresponding author upon reasonable request. Data are stored in a controlled access data storage facility at Pablo de Olavide University (https://jazmin.upo.es/bscw/bscw.cgi, accessed on 22 December 2024).

## Supplementary Materials

The following supporting information can be downloaded at https://www.mdpi.com/article/10.3390/antiox14020215/s1: Figure S1: Treatment effect on superoxide anon levels in all fibroblast lines. Figure S2: Treatment effect on intracellular iron accumulation in all fibroblast lines. Figure S3: Analysis of intracellular iron in control (C1) and mutant (P1, P2, P3) fibroblasts with and without cDNA complementation (+r*GFM1*), detected through Prussian Blue staining. Figure S4: Treatment effect on lipofuscin accumulation, detected by autofluorescence, in all fibroblast lines. Figure S5: Representative images of magnification of electron microscopy images of control (C2) and mutant (P2) fibroblasts. Figure S6: Treatment effect on iron metabolism in all fibroblast lines. Figure S7: Treatment effect on mitochondrial iron content in all fibroblast lines. Figure S8: Treatment effect on lipid peroxidation in all fibroblast lines. Figure S9: Treatment effect on sensitivity of erastin-induced ferroptosis in all fibroblast lines. Figure S10: Galactose drug screening.

## Abbreviations

BPAN	β-propeller Protein-Associated Neurodegeneration
BSA	Bovine Serum Albumine
DAPI	4′,6-diamino-2-phenylindole
DMEM	Dulbecco’s Modified Eagle’s Medium
DMSO	dimethyl sulfoxide
DMT1	divalent metal transporter 1
EF-G1	mitochondrial translation elongation factor G1
FBS	Fetal Bovine Serum
Fe^2+^	ferrous iron
Fe^3+^	ferric iron
FPN	ferroportin
Ft	ferritin
FXN	frataxin
GFM1	G elongation Factor Mitochondrial 1
GPX4	glutathione peroxidase 4
GSH	reduced glutathione
HBSS	Hank’s Balanced Salt Solution
HRP	horseradish peroxidase
ICP-MS	inductively coupled plasma mass spectrometry
iNs	induced neurons
IRP1	iron response protein 1
ISCU	iron sulfur cluster assembly scaffold protein
LHON	Leber’s Hereditary Optic Neuropathy
LIP	labile iron pool
LYRM4	LYR motif-containing protein 4
Mfrn1	mitoferrin1
Mfrn2	mitoferrin2
MnSOD	manganese superoxide
MOI	multiplicity of infection
mtACP	mitochondrial acyl carrier protein
mtDNA	mitochondrial DNA
mtETC	mitochondrial electron transport chain
Mt-Ft	mitochondrial ferritin
mtUPR	mitochondrial Unfolded Protein Response
NBIA	Neurodegeneration with Brain Iron Accumulation
NCOA4	nuclear receptor coactivator 4
nDNA	nuclear DNA
NFS1	NFS1 cysteine desulfurase
Nrf2	nuclear respiratory factor 2
OMM	outer mitochondrial membrane
PANK2	pantothenate kinase 2
PBS	phosphate buffer saline
PFA	paraformaldehyde
PI	propidium iodide
PKAN	Pantothenate Kinase-Associated Neurodegeneration
PLAN	PLA2G6-Associated Neurodegeneration
ROS	reactive oxygen species
STEAP3	metalloreductase six-transmembrane epithelial antigen of prostate 3
TfR1	transferrin receptor protein 1
