# Supplementary material for: Polydatin and Nicotinamide Prevent Iron Accumulation and Lipid Peroxidation in Cellular Models of Mitochondrial Diseases
Source: Antioxidants (Basel). 2025 Feb 13;14(2):215. doi: 10.3390/antiox14020215 (PMC11851670; doi:10.3390/antiox14020215)
Supplement: Supplementary file 1 [file antioxidants-14-00215-s001.zip › antioxidants-3414945-supplementary.pdf]

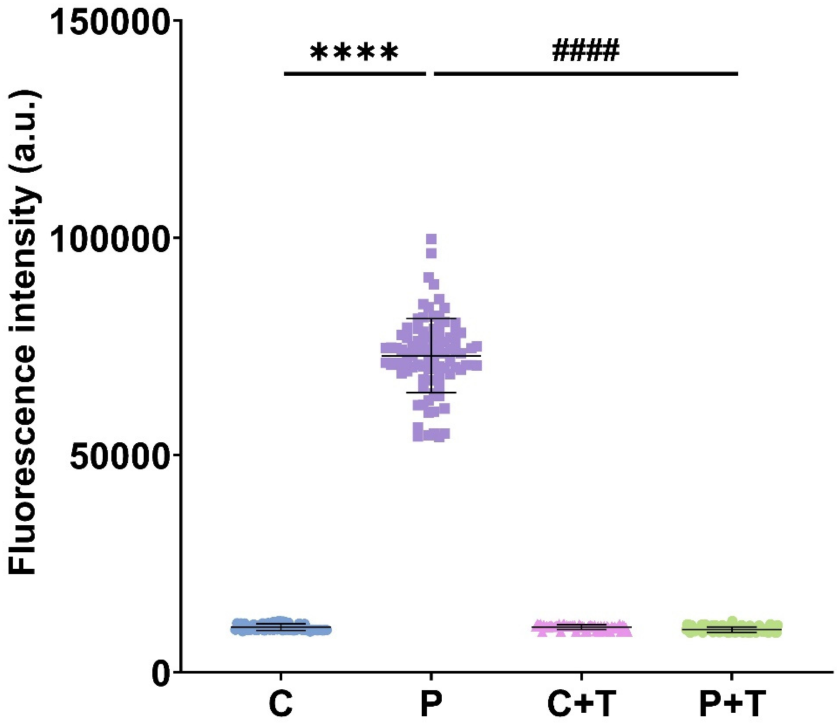

Figure S1. Treatment effect on superoxide anion levels in all fibroblast lines. Comparison of superoxide anion levels in untreated and treated (+T) control and patient-derived fibroblasts. Data represent the mean ± SD from at least three independent cell lines per group. C = C1, C2. P = P1, P2, P3. ****$p<0.0001$ between control and mutant fibroblasts. ####$p<0.0001$ between untreated and treated patients' fibroblasts. a.u.: arbitrary units.

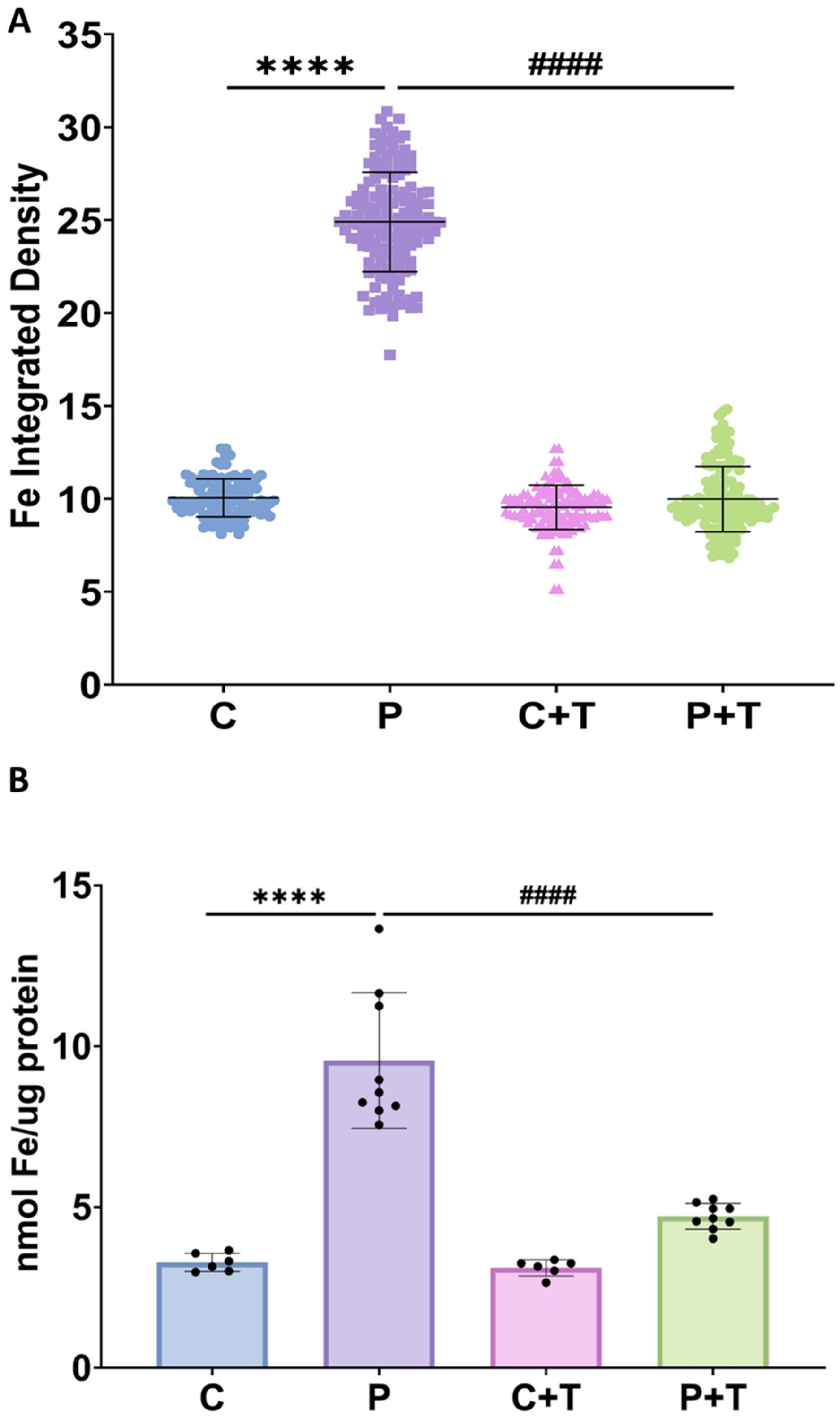

Figure S2. Treatment effect on intracellular iron accumulation in all fibroblast lines. **A.** Comparison of Fe Integrated Density, quantified from Prussian Blue staining, in untreated and treated (+T) control and patient-derived fibroblasts. **B.** Comparison of iron levels, detected by ICP-MS, in untreated and treated (+T) control and patient-derived fibroblasts. Data represent the mean ± SD from at least three independent cell lines per group. C = C1, C2. P = P1, P2, P3. ****$p<0.0001$ between control and mutant fibroblasts. $^{####}p<0.0001$ between untreated and treated patients' fibroblasts. a.u.: arbitrary units.

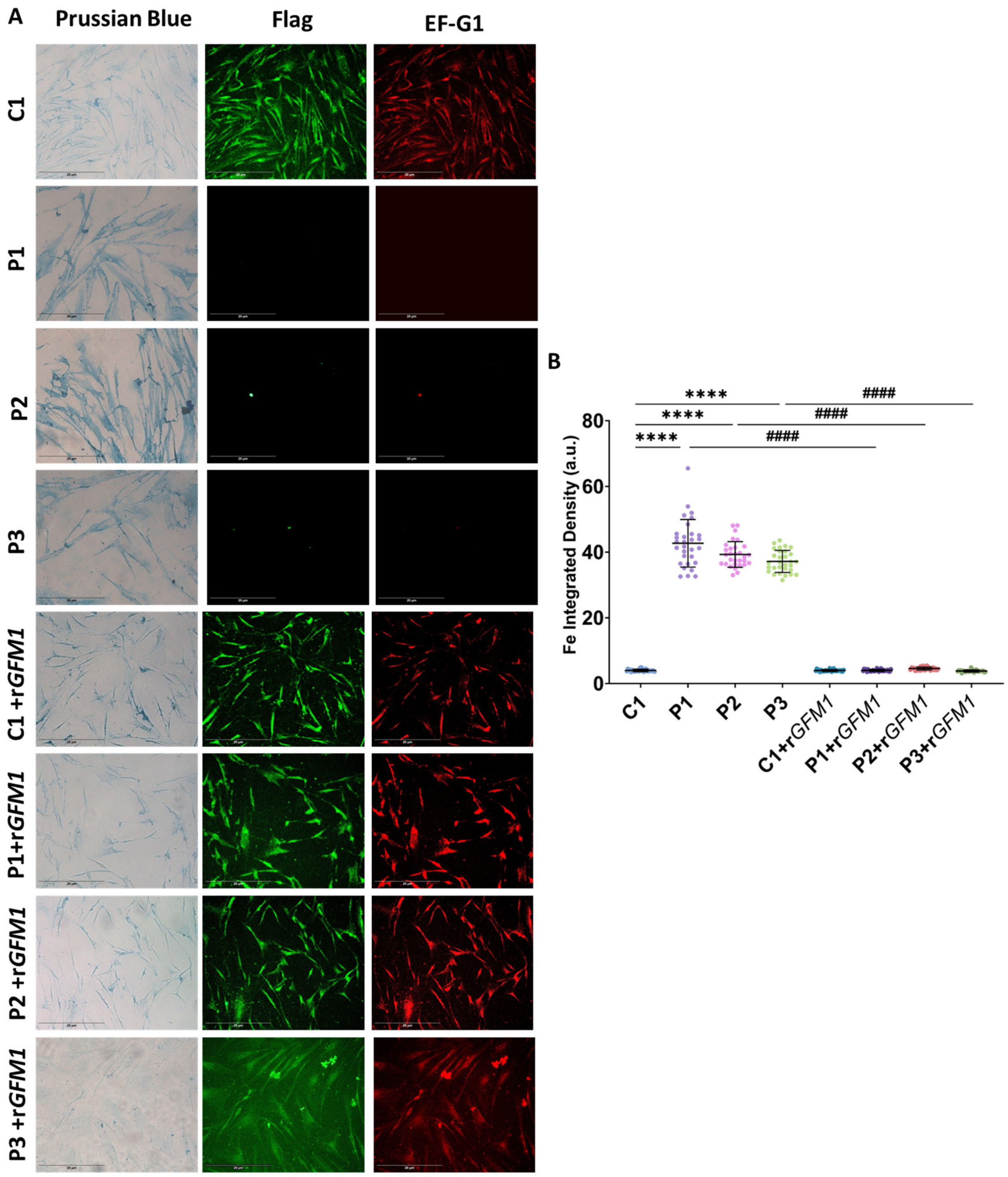

Figure S3. Analysis of intracellular iron in control (C1) and mutant (P1, P2, P3) fibroblasts with and without cDNA complementation (+*rGFM1*), detected through Prussian blue staining. (**A**) Representative images of Prussian blue staining and immunofluorescence with anti-Flag and anti-GFM1 antibodies. Scale bar = 20 µm. (**B**) Quantification of integrated iron density. Data represent the mean ± SD of three independent experiments (at least 30 images were taken from each condition and experiment). Number of replicates = 3. ****$p<0.0001$ between control and mutant fibroblasts. ####$p<0.0001$ between mutant fibroblasts without and with cDNA complementation.

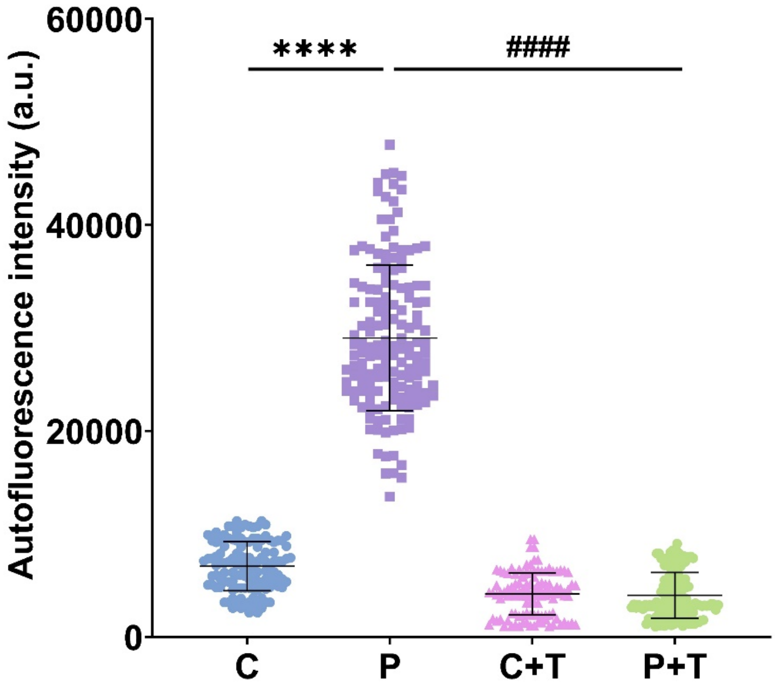

Figure S4. Treatment effect on lipofuscin accumulation, detected by autofluorescence, in all fibroblast lines. Data represent the mean ± SD from at least three independent cell lines per group. C = C1, C2. P = P1, P2, P3. ****p<0.0001 between control and mutant fibroblasts. ####p<0.0001 between untreated and treated patients' fibroblasts. a.u.: arbitrary units.

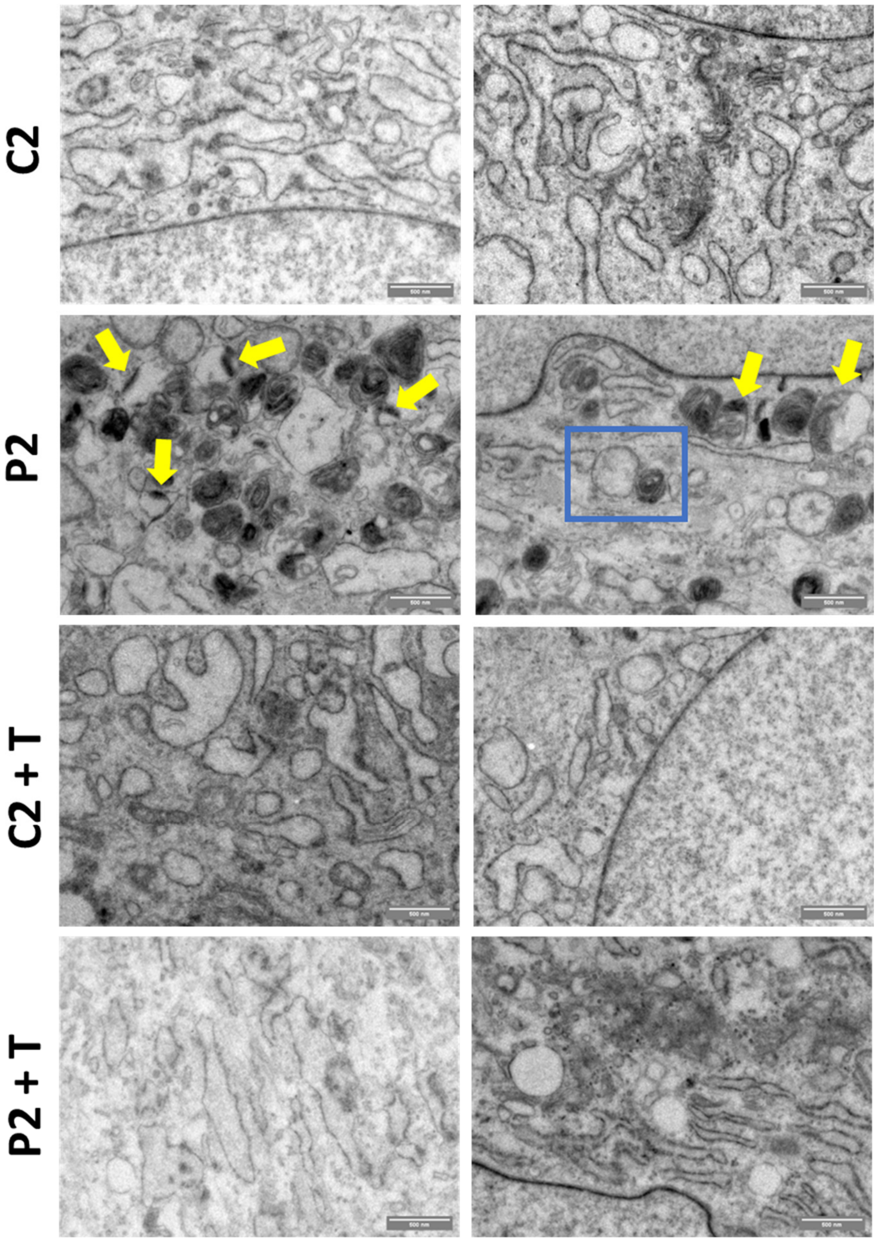

Figure S5. Representative images of magnification of electron microscopy images of control (C2) and mutant (P2) fibroblasts. Yellow arrows: lipofuscin granules forming inside the mitochondrial. Blue box: expulsion of a lipofuscin granule from the mitochondria into the cytosol: Scale bar = 500 nm.

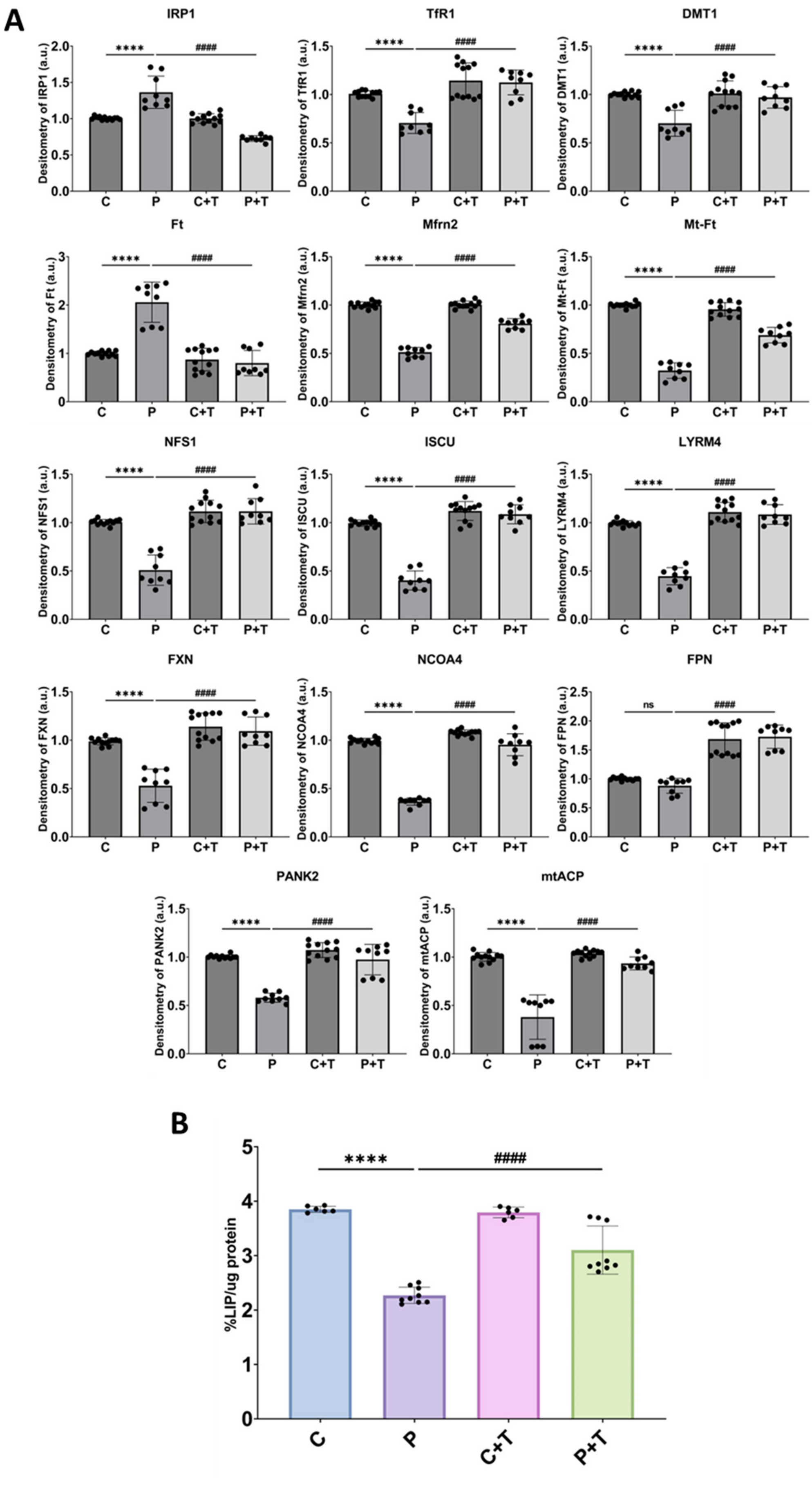
A
IRP1
Densitometry of IRP1 (a.u.)
TfR1
Densitometry of TfR1 (a.u.)
DMT1
Densitometry of DMT1 (a.u.)
Ft
Densitometry of Ft (a.u.)
Mfrn2
Densitometry of Mfrn2 (a.u.)
Mt-Ft
Densitometry of Mt-Ft (a.u.)
NFS1
Densitometry of NFS1 (a.u.)
ISCU
Densitometry of ISCU (a.u.)
LYRM4
Densitometry of LYRM4 (a.u.)
FXN
Densitometry of FXN (a.u.)
NCOA4
Densitometry of NCOA4 (a.u.)
FPN
Densitometry of FPN (a.u.)
ns
PANK2
Densitometry of PANK2 (a.u.)
mtACP
Densitometry of mtACP (a.u.)
****
####
C
P
C+T
P+T
B
%LIP/ug protein

Figure S6. Treatment effect on iron metabolism in all fibroblast lines. **A.** Densitometry of Western Blot data of all analyzed proteins. **B.** LIP levels. Data represent the mean ± SD from at least three independent cell lines per group. C = C1, C2. P = P1, P2, P3. ****p<0.0001 between control and mutant fibroblasts. ####p<0.0001 between untreated and treated patients' fibroblasts. a.u.: arbitrary units.

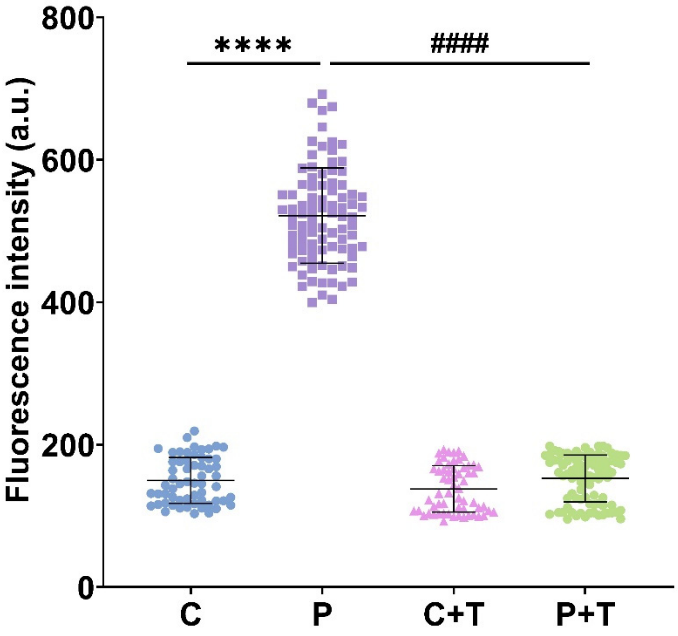

Figure S7. Treatment effect on mitochondrial iron content in all fibroblast lines. Data represent the mean ± SD from at least three independent cell lines per group. C = C1, C2. P = P1, P2, P3. ****p<0.0001 between control and mutant fibroblasts. ####p<0.0001 between untreated and treated patients' fibroblasts. a.u.: arbitrary units.

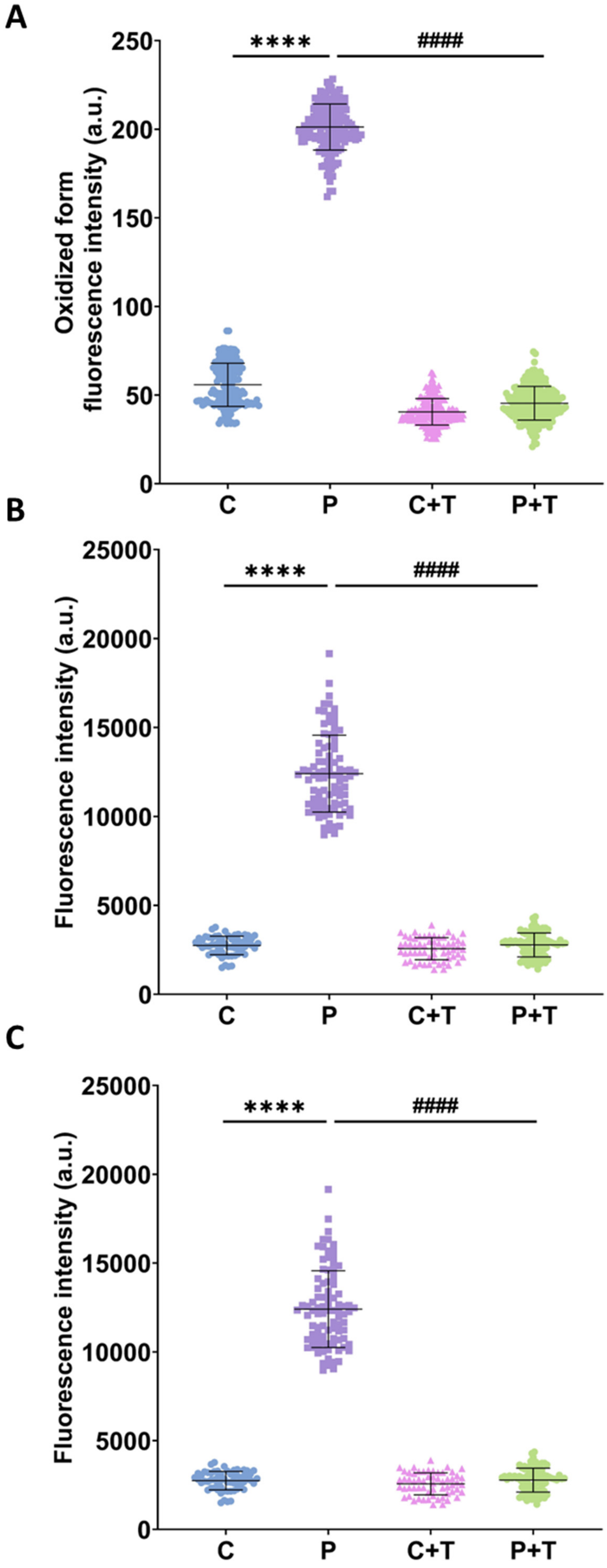

A
****
####
Oxidized form fluorescence intensity (a.u.)
250
200
150
100
50
0
C
P
C+T
P+T
B
****
####
Fluorescence intensity (a.u.)
25000
20000
15000
10000
5000
0
C
P
C+T
P+T
C
****
####
Fluorescence intensity (a.u.)
25000
20000
15000
10000
5000
0
C
P
C+T
P+T

Figure S8. Treatment effect on lipid peroxidation in all fibroblast lines. **A.** Cellular lipid peroxidation, detected by Bodipy® 581/591 C11 staining. **B.** GSH levels, detected by ThiolTracker^{TM} Violet staining. **C.** Mitochondrial lipid peroxidation, detected by MitoPeDPP® staining. Data represent the mean ± SD from at least three independent cell lines per group. C = C1, C2. P = P1, P2, P3. ****p<0.0001 between control and mutant fibroblasts. $^{####}$p<0.0001 between untreated and treated patients' fibroblasts. a.u.: arbitrary units.

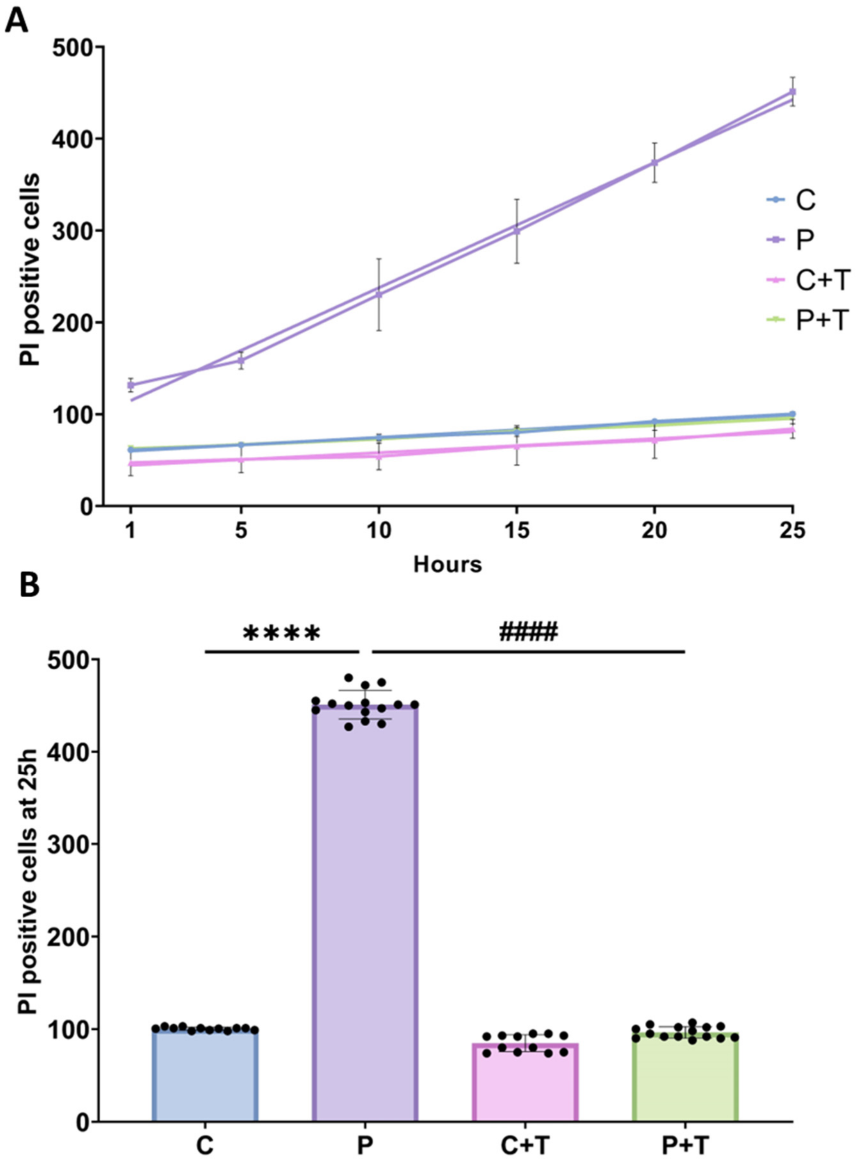

Figure S9. Treatment effect on sensitivity of erastin-induced ferroptosis in all fibroblast lines. **A.** PI positive cells over the period of 25 hours. **B.** PI positive cells at 25 hours. Data represent the mean ± SD from at least three independent cell lines per group. C = C1, C2. P = P1, P2, P3. ****p<0.0001 between control and mutant fibroblasts. $^{####}$p<0.0001 between untreated and treated patients' fibroblasts. a.u.: arbitrary units.

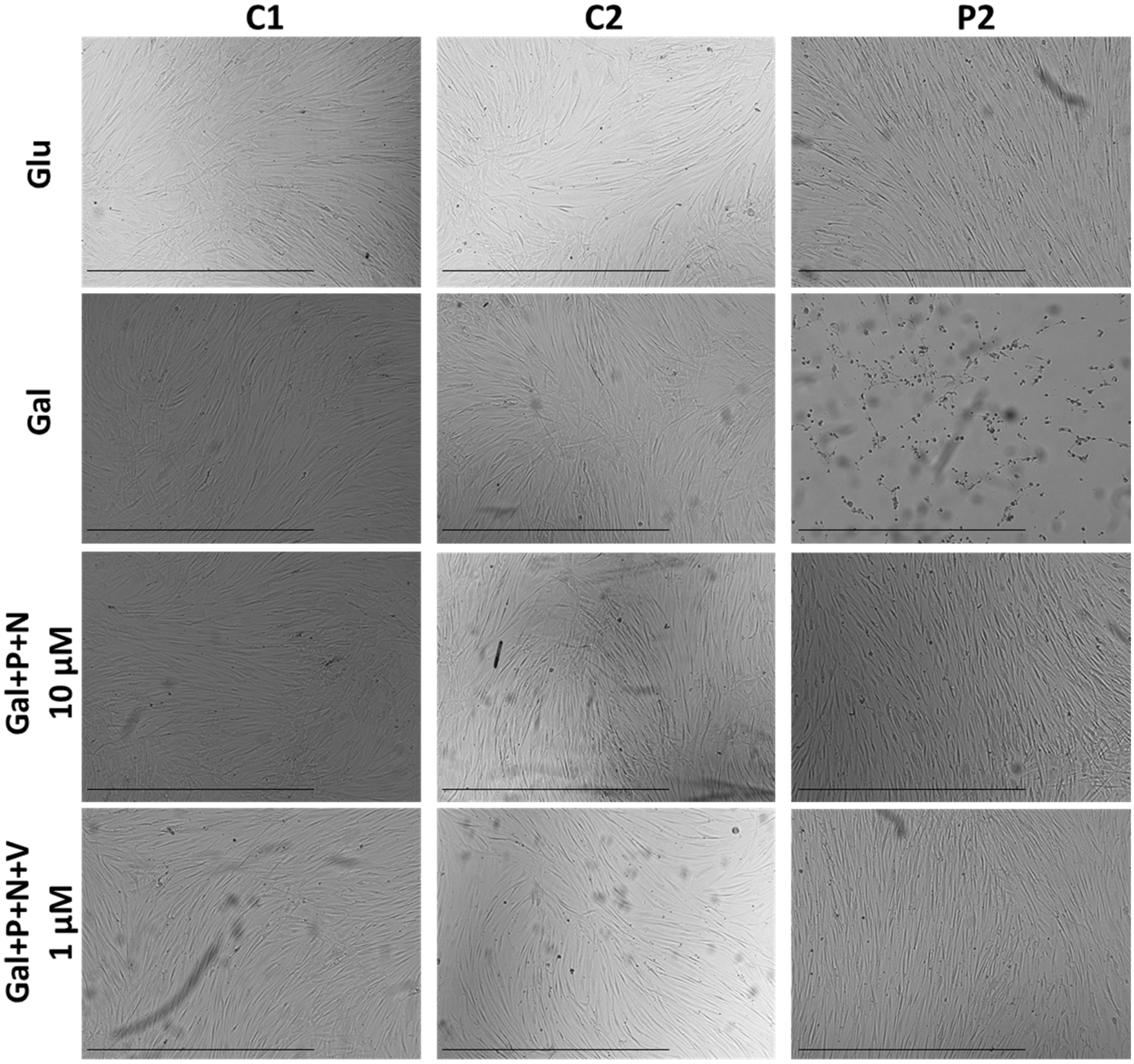

Figure S10. Galactose drug screening. Cells were initially seeded in glucose medium and treated with several combinations of polydatin (P), nicotinamide (N), and vitamin E (V). After 3 days, the glucose medium was changed to galactose medium, and treatments were refreshed. Images were obtained using the BioTek Cytation 1 Cell Imaging Multi-Mode Reader. Scale bar = 1000 µm. Glu: glucose; Gal: galactose; P: polydatin; N: Nicotinamide; V: vitamin E.
